# Lewis Acid‐Triggered Spatiotemporally Controllable Ring Opening in a Classic Rhodamine Featuring Φ = 95% Emission

**DOI:** 10.1002/smsc.202500522

**Published:** 2026-01-22

**Authors:** Quanchun Sun, Liancheng He, Tao Wang, Haiyan Cui, Xinping Wang

**Affiliations:** ^1^ State Key Laboratory of Coordination Chemistry School of Chemistry and Chemical Engineering Collaborative Innovation Center of Advanced Microstructures Nanjing University Nanjing 210023 P. R. China; ^2^ State Key Laboratory of Organometallic Chemistry Shanghai Institute of Organic Chemistry Chinese Academy of Science Shanghai 200032 P. R. China; ^3^ College of Sciences Nanjing Agricultural University Nanjing 210095 P. R. China

**Keywords:** emissions, Lewis acid, ring opening of rhodamine, superior quantum yields

## Abstract

Rhodamine derivatives, as a prominent class of fluorophores, have become indispensable in advanced material engineering and biomedical research due to their exceptional photostability and tunable optical characteristics. However, their practical implementation faces fundamental challenges: conventional proton‐mediated spirolactone ring opening mechanisms severely compromise fluorescence performance, while conventional structural optimization approaches remain synthetically demanding with limited efficacy. We hereby present a novel Lewis acid‐assisted activation strategy that enables reversible spirolactone ring opening in classical rhodamine systems. This innovative approach achieves remarkable fluorescence enhancement characterized by superior quantum yields (up to 95%) and prolonged excited state lifetimes. Notably, the Lewis acid coordination establishes precise photocontrol over the ring opening process. This breakthrough represents the first demonstration of a nondestructive activation pathway for rhodamine fluorophores, effectively converting the nonemissive spirolactone form into highly luminescent Lewis acid complexes while maintaining molecular integrity.

## Graphical abstract

This work reports a breakthrough in rhodamine activation via an elegant Lewis acid coordination strategy. It induces ring opening in spirolactone rhodamine TMR, generating exceptional fluorescence (quantum yields up to 95%) without destructive modifications or structural modification. The method preserves full photocontrol, reversibility, and real‐time tunability, thereby paving the way for advanced fluorescent material design.

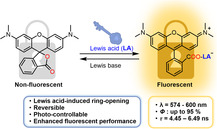

## 1 Introduction

**Scheme 1:**
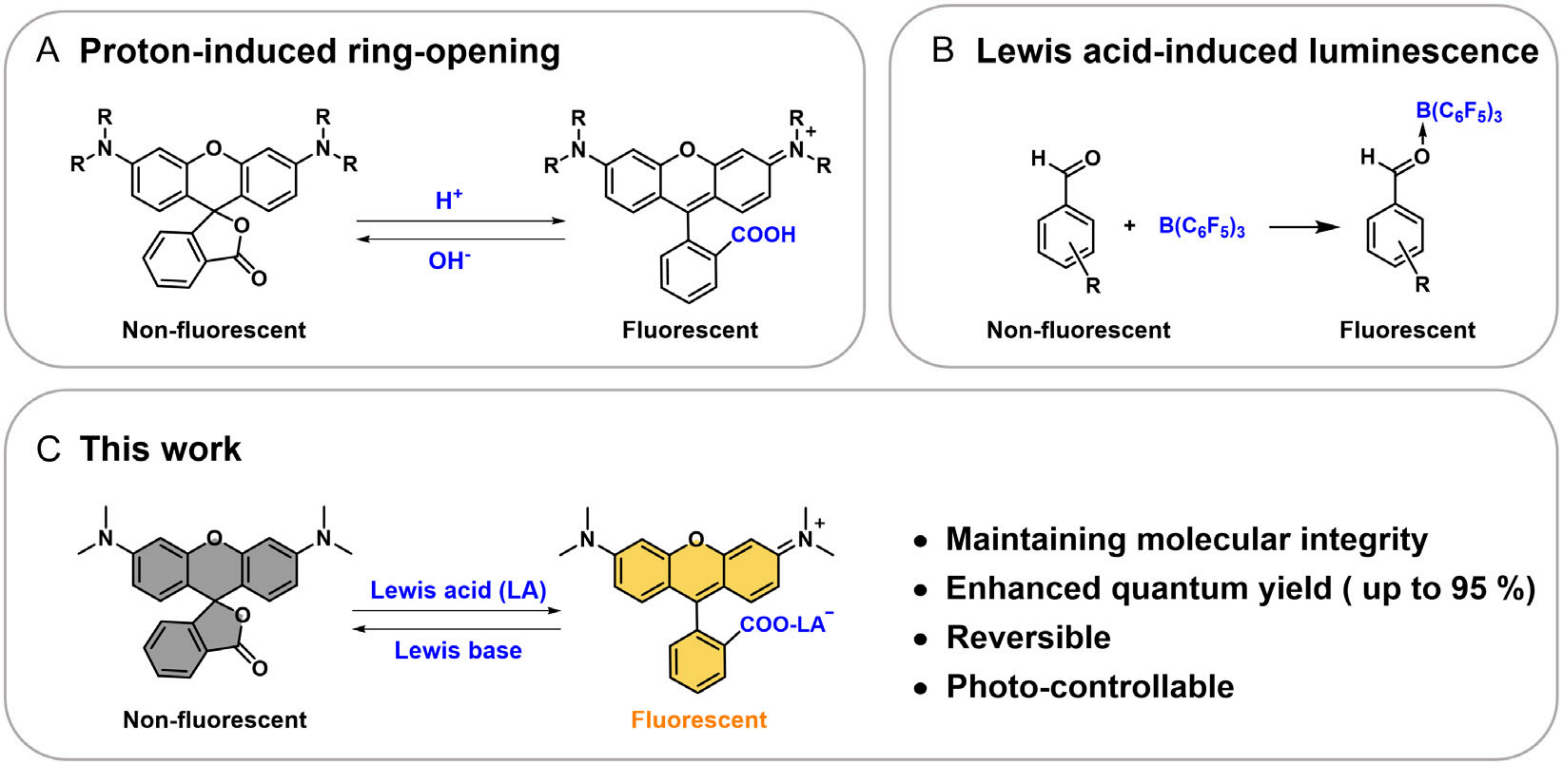
LA‐ and proton‐induced luminescence. A) Proton‐induced ring opening of rhodamines and the reverse reactions. B) BCF‐induced luminescence of aldehydes. C) LA‐induced ring opening of TMR and the reverse reactions.

Rhodamines, as classic fluorophores, are widely used in materials science, biomedicine, and environmental monitoring, owing to their exceptional photostability, high brightness, and excellent biocompatibility.^[^ ^1^, ^2^, ^3^, ^4^, ^5^, ^6^ ^]^ These dyes typically operate via a proton‐induced ring opening mechanism, reversibly switching between nonfluorescent spirolactone (closed ring) and fluorescent cationic (open ring) states (**Scheme** **1**A). However, conventional proton‐activated rhodamine systems face inherent limitations, including low quantum yield, poor tissue penetrability, low signal‐to‐noise ratios (SNRs), and susceptibility to light scattering, significantly restricting their applicability. To address these challenges, extensive efforts have focused on structural modifications,^[^ ^7^, ^8^, ^9^, ^10^, ^11^, ^12^, ^13^, ^14^, ^15^, ^16^, ^17^ ^]^ such as i) π‐conjugation extension, ii) xanthene oxygen substitution, iii) intramolecular charge transfer (ICT) enhancement via donor/acceptor moieties, and iv) lactam/sultam replacement of the lactone group.^[^ ^18^, ^19^ ^]^ Nevertheless, these strategies often require time‐consuming synthesis and stringent reaction conditions. Moreover, many modified rhodamine analogs demonstrate marginal improvements in fluorescence quantum yields, photostability, or brightness.^[^ ^20^ ^]^ Thus, developing a facile and universally applicable methodology to enhance the fluorescence performance of rhodamines, without intricate structural redesign, remains a critical challenge in molecular photonics.

Main‐group Lewis acids (LAs) have attracted considerable research interest owing to their versatile applications in organic synthesis, catalysis, and materials science.^[^ ^21^, ^22^, ^23^, ^24^, ^25^, ^26^, ^27^, ^28^ ^]^ Their emerging roles in photoelectric materials have witnessed remarkable advancements in recent decades. Through doping strategies, LAs can effectively modulate the band gaps and absorption properties of organic semiconductors, leading to narrowed optical bandgaps and red‐shifted absorption spectra to even near‐infrared (NIR) region.^[^ ^29^, ^30^, ^31^, ^32^, ^33^ ^]^ Strong LAs (e.g., B(C_6_F_5_)_3_, BCF) exhibit additional functionalities in optoelectronics, such as enhancing electrochemiluminescence (ECL) in donor‐acceptor (D‐A) semiconductors^[^ ^34^ ^]^ and enabling multicolor emission through tunable complexation.^[^ ^35^ ^]^ Intriguingly, even nonemissive aldehydes can be rendered photoluminescent in the solid state upon BCF binding^[^ ^30^ ^]^ (Scheme 1B). Moreover, triphenylaminoformaldehyde‐BCF complexes display piezochromic luminescence, exhibiting mechano‐responsive blue shifted and intensified emission.^[^ ^36^ ^]^ On the other hand, the acidity of LAs can also be determined by using fluorescence.^[^ ^37^, ^38^, ^39^ ^]^ In recent years, our research group has focused on investigating single‐electron transfer (SET) interactions between LAs and organic molecules. Through the LA‐coupled electron transfer (LACET) strategy, we have successfully synthesized a series of radical compounds and extended their applications to diverse fields, including dynamic chemistry,^[^ ^40^, ^41^ ^]^ carbene chemistry,^[^ ^42^ ^]^ small‐molecule activation,^[^ ^43^ ^]^ and photothermal conversion systems.^[^ ^44^ ^]^ Lewis bases also provide a means to tune the photophysical properties of D‐A systems. Recently, Silva, Takeda, and their coworkers have demonstrated that the fluoride anion (F^−^) can induce red shifts in both the absorption and emission of a D‐A system via narrowing of the HOMO‐LUMO energy gap.^[^ ^45^ ^]^

The ring opening of the rhodamine lactone scaffold is the basis for its application in fluorescent dyes. Although, in the prior reports, it was well‐documented that inorganic LAs (e.g., Li^+^, Cu^2+^, and Mn^2+^) effectively trigger this ring opening, leading to chromogenic and fluorogenic responses,^[^ ^46^, ^47^, ^48^ ^]^ this paradigm is predominantly confined to metal‐sensing applications with little enhancement in fluorescence performance, while organic LAs present a highly attractive alternative owing to their structural diversity, tunability, and potential for enhanced compatibility with organic systems. We hypothesized that organic LAs may also induce the ring opening of rhodamines and probably enhance optical properties. Herein, we report a LA‐induced ring opening strategy, converting nonfluorescent spirolactone TMR into highly emissive adducts using Lewis superacids Al(OR^F^)_3_ (R^F^ = C(CF_3_)_3_), BCF and SiR_3_^+^ (with BAr^F^_4_^−^ counter anion; R = CH(CH_3_)_2_, Ar^F^ = C_6_F_5_) (Scheme 1C). The compounds were comprehensively characterized by single‐crystal X‐ray diffraction, NMR spectroscopy, UV‐Vis absorption, and fluorescence emission spectroscopy. Unlike proton‐activated derivatives, the LA‐rhodamine open ring adducts exhibited significantly enhanced photoluminescence quantum yields and prolonged excited‐state lifetimes. Crucially, this strategy requires no core structural modifications, retains photocontrollable reversibility, and offers a streamlined route to high‐performance fluorophores.

## 2 Results and Discussion

### 2.1 Synthesis and Crystal Structures

**Figure 1:**
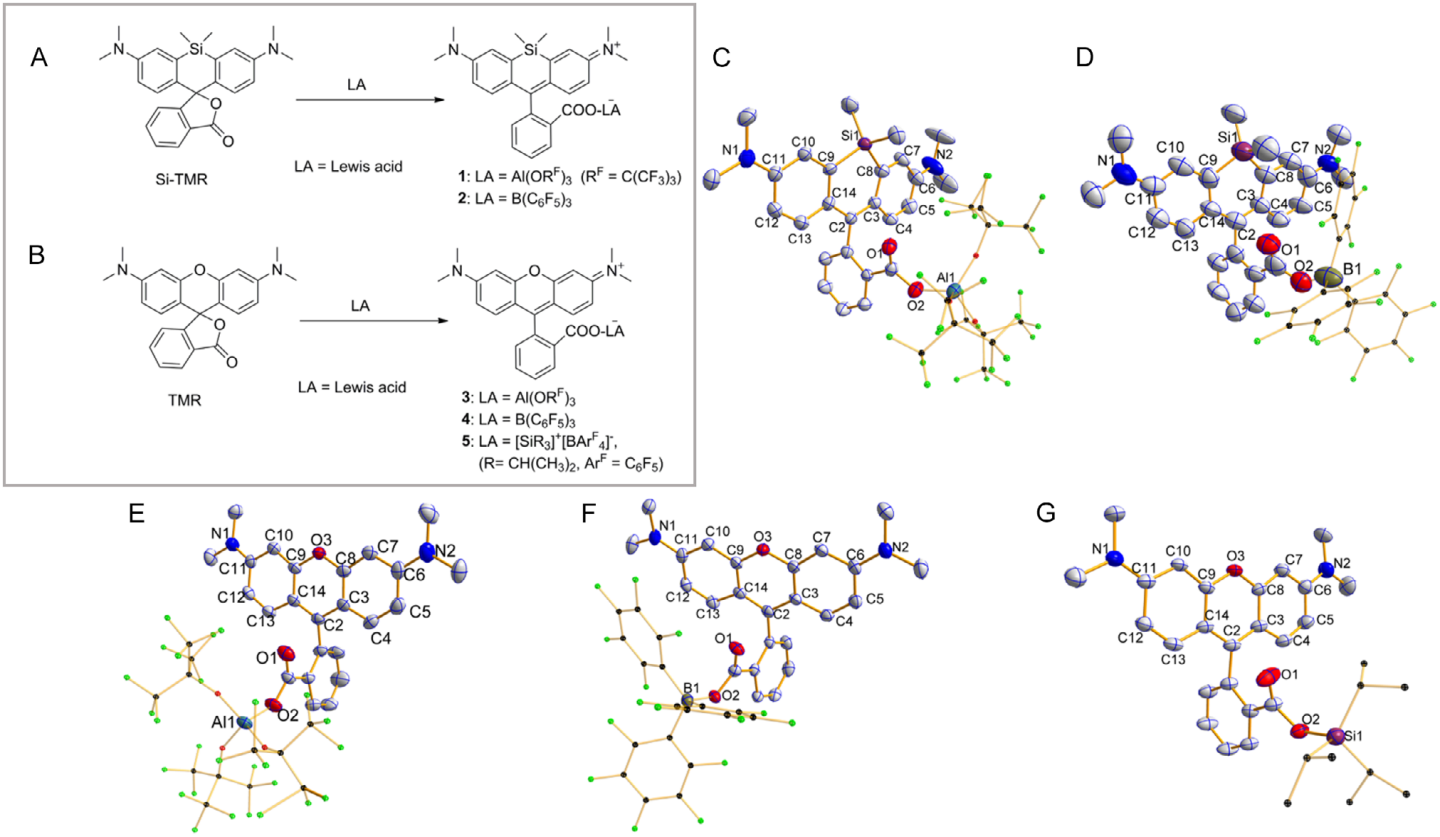
LA‐induced ring opening processes of rhodamines and crystal structures of **1**–**5**. A) Reactions of Si‐TMR with LAs. B) Reactions of TMR with LAs. C–D) Thermal ellipsoid drawings of **1** and **2** with 50% probability. E–G) Thermal ellipsoid drawings of **3**–**5** with 50% probability. LA moieties are shown in ball‐and‐stick model, and hydrogen atoms and BAr^F^_4_^−^ anion in **5** were omitted for clarity.

The spirolactone precursors Si‐TMR and TMR were synthesized following established protocols.^[^ ^49^, ^50^ ^]^ When subjected to LA activation, Si‐TMR underwent smooth coordination with BCF or Al(OR^F^)_3_ in toluene at ambient conditions, producing air‐stable blue (**1**) and green (**2**) crystalline complexes, respectively (**Figure** **1**A). Parallel treatment of TMR with stoichiometric Al(OR^F^)_3_,^[^ ^51^ ^]^ BCF, or SiR_3_^+^•BAr^F^_4_^−^^[^ ^52^ ^]^ in aromatic solvents (toluene/fluorobenzene) yielded three distinct crystalline derivatives (**3**–**5**) with characteristic red‐to‐purple coloration (Figure 1B). Remarkably, all resultant complexes (**1**–**5**) exhibited exceptional stability against atmospheric moisture and oxygen, maintaining structural integrity both in solid state and solution under ambient storage conditions.

High‐quality single crystals of complexes **1**–**5** were successfully grown via slow crystallization from mother liquor at −20 °C. X‐ray diffraction analysis revealed distinct crystallographic characteristics: compounds **1** and **3**–**5** adopt triclinic P1 symmetry, whereas **2** crystallizes in the monoclinic *P*2_1_/c space group.^[^ ^53^ ^]^ Structural determination unambiguously confirmed LA‐mediated ring opening through coordination to carbonyl oxygen atoms, with full structural illustrations provided in Figure 1C–G, and selected parameters are given in Table S1–S4, Supporting Information.

**Scheme 2:**
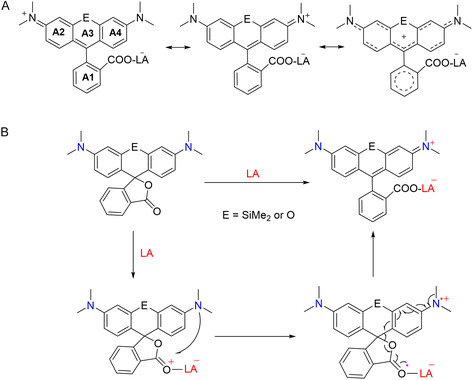
Resonance structures of rhodamine–LA compounds and proposed ring opening mechanism. A) Resonance structures for rhodamines–LA. B) Proposed mechanism (Path A) for the ring opening process of rhodamine spirolactones with Lewis superacid.

Notably, steric effects dictate conformational variations across the series. In silicon‐containing complexes **1**–**2**, since the conjugation systems are partially separated by the sp^3^ hybrid silicon atom in SiMe_2_ groups, the pyronin parts are slightly curved (Figure 1C–D), which show a dihedral angle of 83.03° in 1 and 76.47° in **2** with ring A1 (A1–A4 are shown in **Scheme** **2**A). The CO_2_X (X = Al, B) moieties deviate significantly from planarity with benzene ring A1, while the CO_2_X (X = Al, B, Si) moieties in aluminum‐, boron‐ and silicon‐coordinated derivatives **3**–**5** are nearly coplanar with their A1 planes, which exhibit dihedral angles of 87.53°, 72.21°, and 82.8° with their corresponding pyronin planes (Figure 1E–G), respectively. For **1** and **2**, the average bond lengths (1.35–1.37 Å) of C4–C5, C7–C8, C9–C10, and C12–C13 are notably shorter than the average value of other bond lengths (1.40–1.43 Å) in the rings A4 and A2, showing quinoidal characters. In addition, the bond lengths C2–C3 and C2–C14 (1.39–1.41 Å) in **3**–**5** are nearly equal and also between the bond lengths of C—C single bond and C=C double bond. The similar situation occurs in **3**–**5**. Compounds **1**–**5** can be best described as the resonance structures shown in Scheme 2A.

### 2.2 Single‐Electron Transfer (SET) Mechanism

Two distinct SET pathways were proposed for the formation of complexes **1**–**5**. Mechanism A (Scheme 2B) initiates through LA coordination to the carbonyl oxygen, inducing intramolecular SET from the pyronin π‐system to the carboxylate fragment. This electronic redistribution triggers spirolactone ring opening, generating a fluorescent zwitterionic structure with restored conjugation. Mechanism B (Figure S2, Supporting Information) involves intermolecular SET between a Lewis superacid and the closed ring rhodamine, forming a metastable radical ion pair intermediate [(Si‐)TMR^•+^LA^•‐^].^[^ ^44^, ^54^ ^]^ Subsequent radical coupling at the carbonyl oxygen facilitates homolytic C_spiro_—O bond cleavage, followed by sequential SET events to stabilize the final open ring product. Critical evidence for these SET processes emerged from in situ EPR spectroscopy of the TMR/BCF reaction system. Distinct radical signals observed across cryogenic to ambient temperatures (Figure S1, Supporting Information) validates the SET pathway.

### 2.3 Photophysical Characteristics

**Figure 2:**
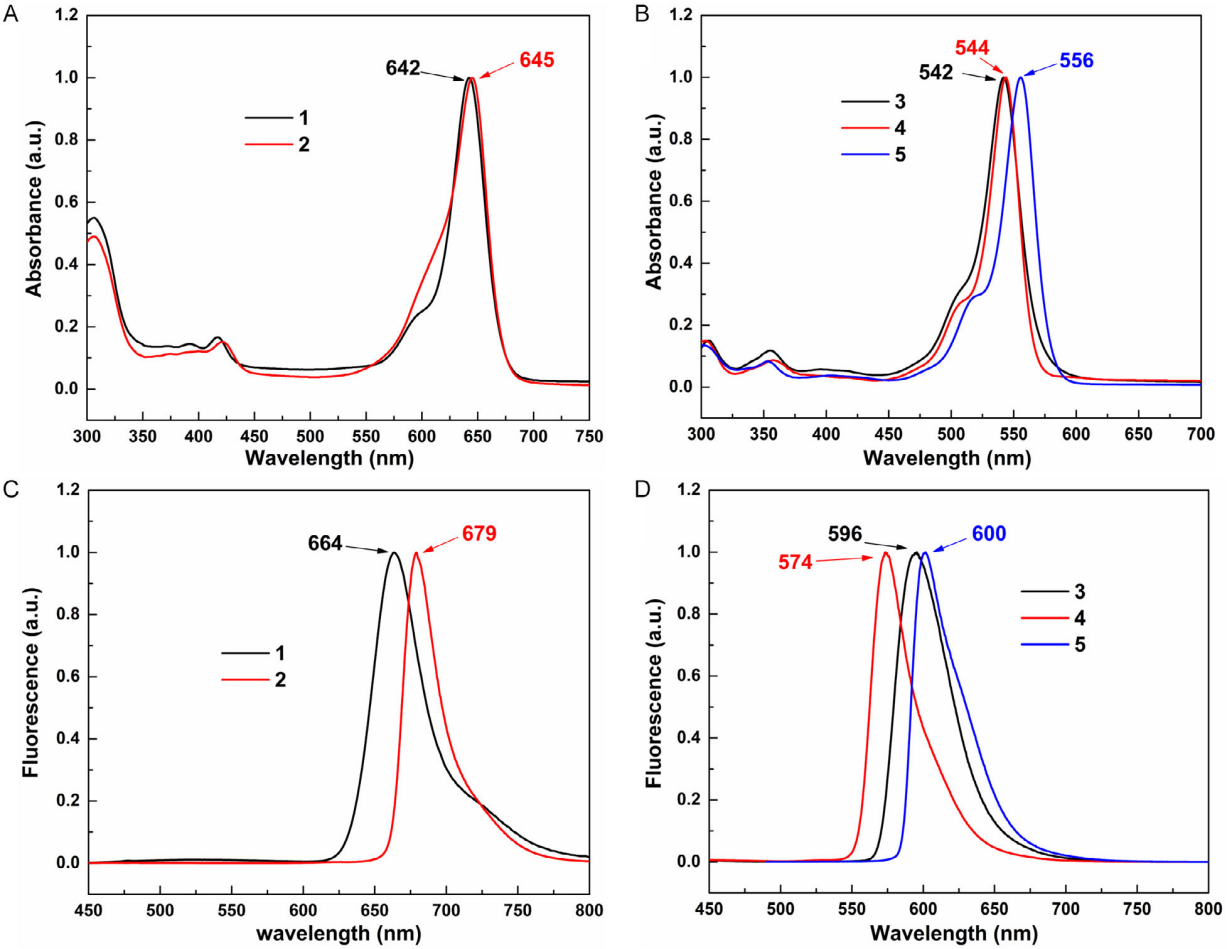
Optical spectra. A) UV‐Vis absorption (*λ*_abs_) spectra of **1**–**2** in toluene at room temperature. B) UV‐Vis absorption (*λ*_abs_) spectra of **3**–**5** in toluene at room temperature. C) Fluorescence emission (*λ*_em_) spectra of **1**–**2** in toluene at room temperature. D) Fluorescence emission (*λ*_em_) spectra of **3**–**5** in toluene at room temperature.

The photophysical characteristics of complexes **1**–**5** were systematically investigated to elucidate structure‐property relationships (**Figure** **2** and **Table** **1**). For silicon‐containing systems (**1**–**2**), toluene solutions exhibited intense absorption bands at 642 nm (**1**) and 645 nm (**2**) (Figure 2A), demonstrating 319–322 nm redshifts relative to their spirolactone precursor (323 nm, Figure S3, Supporting Information). These spectral profiles closely matched proton‐activated Si‐TMR (*λ*_abs_ = 643 nm),^[^ ^55^ ^]^ confirming successful ring opening. Fluorescence analysis revealed distinct emission at 664 (**1**) and 679 nm (**2**) (Figure 2C), comparable with proton‐induced analog (*λ*_em_ = 662 nm).^[^ ^55^ ^]^ Comparative photometric analysis uncovered threefold enhancements: i) 22–34 nm Stokes shifts (vs. 19 nm for proton‐activated Si‐TMR), ii) prolonged lifetimes (*τ* = 8.48 ns for **2** vs. 5.04 ns, Figure S17, Supporting Information), and iii) maintained quantum yields (Φ = 36.09–41.22% vs. 41%). In O‐containing systems (**3**–**5**), the UV‐Vis absorption maxima (542–556 nm, Figure 2B) are similar to the absorption wavelength of TMR in ring opening form (*λ*_abs_ = 548 nm),^[^ ^55^ ^]^ and 238–251 nm redshifted compared with their precursor spirolactone form TMR (*λ*_abs_ = 305 nm, Figure S4, Supporting Information). The emissions of **3**–**5** emerged at 574–600 nm (Figure 2D), slightly redshifted from proton‐opened TMR (572 nm).^[^ ^55^ ^]^ Quantum leap in performance was observed: **3**–**5** achieved record Φ values of 91.69–94.87% (vs. 41% for proton system), 30–56 nm Stokes shifts, and extended *τ* = 4.45–6.49 ns (2.21 ns for proton system). This collective enhancement establishes LA coordination as a superior activation strategy over conventional protonation.

**Table 1:** Optical properties of **1**–**5**.

Compounds	*λ*_abs_ [nm]	*λ*_em_ [nm]	Δ*λ* [nm]	Φ [%]	*τ* [ns]
**1**	642	664	22	41.22	4.18
**2**	645	679	34	36.09	8.48
**3**	542	596	56	91.69	6.49
**4**	544	574	30	94.87	4.45
**5**	556	600	44	93.39	6.07

Current strategies for developing O‐rhodamine dyes with high fluorescence performance predominantly rely on π‐extension architectures, annulation‐induced rigidity enhancement, and electron‐withdrawing substituents on the A1 aromatic system.^[^ ^56^, ^57^, ^58^, ^59^, ^60^ ^]^ Notably, our LA‐coordinated systems **3**–**5** demonstrate unprecedented photophysical superiority over these conventional structural modifications, achieving near‐unity quantum yields (Φ = 91–95% vs. 10–88%), and prolonged emission lifetimes (*τ* = 4.45–6.49 ns vs. 0.59–3.84 ns).^[^ ^55^ ^]^ Mechanistic differentiation emerges in fluorescence enhancement pathways. While state‐of‐the‐art TMR derivatives employ steric confinement through N‐adjacent rigid rings to suppress twisted ICT (TICT),^[^ ^61^, ^62^, ^63^ ^]^ compounds **3**–**5** achieve superior performance through electronic modulation without structural alteration. This coordination‐driven approach simultaneously enables i) near‐complete suppression of nonradiative decay (Φ > 91%), ii) enhanced excited‐state stability (*τ* > 4 ns), and iii) enlarged Stokes shifts (30–56 nm) vs proton‐induced analog (24 nm). Critical advancement lies in bypassing the traditional rigidity‐fluorescence approach. Unlike conventional dyes where extended π‐systems compromise quantum yields, our LA activation strategy establishes a new paradigm for developing high‐performance fluorophore.

**Figure 3:**
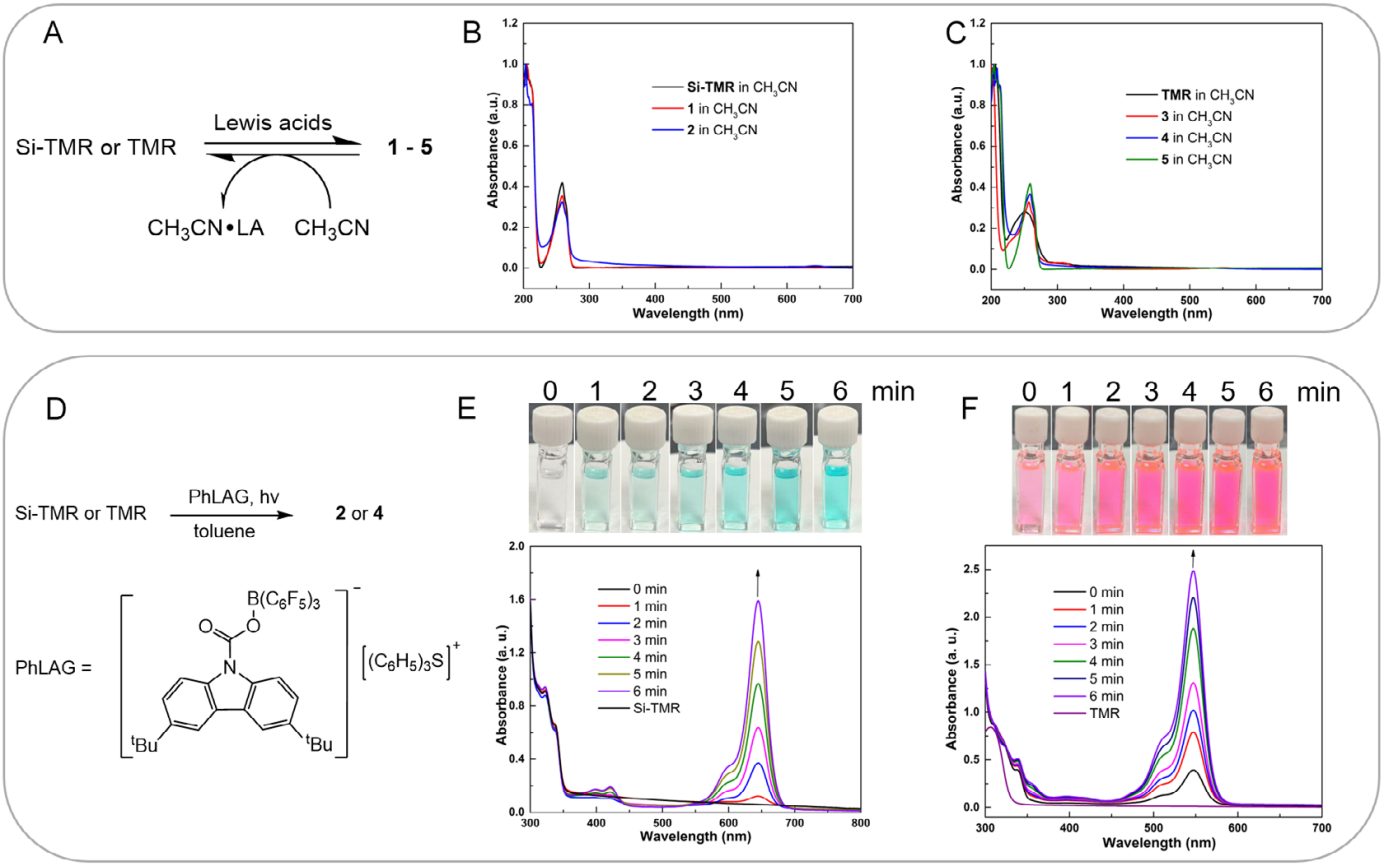
Reversibility and photo‐controllability. A) The reversible process between **1**–**5** and their precursors. B) UV‐Vis absorptions of Si‐TMR and **1**–**2** in CH_3_CN. C) UV‐Vis absorptions of TMR and **3**–**5** in CH_3_CN. D) Photo‐controllable ring opening processes. E) Color changes (top) and UV‐Vis absorptions (below) of Si‐TMR in toluene in the presence of PhLAG by irradiation at 254 nm. F) Color changes (top) and UV‐Vis absorptions (below) of TMR in toluene in the presence of PhLAG by irradiation at 254 nm.

The reversibility and photo responsiveness of the LA‐mediated ring opening process were systematically interrogated through solvent‐ and light‐triggered experiments. Dissolving complexes **1**–**5** in acetonitrile (Lewis base) resulted in immediate fluorescence quenching (**Figure** 3A), concomitant with UV‐Vis spectral reversion to spirolactone signatures (Figure 3B,C). This establishes a dynamic equilibrium between open/closed states, enabling multicycle fluorescence switching through sequential LA/base treatments. The LA induced ring opening processes of spirocyclic Si‐ and TMR have also been found to be photo‐controllable (Figure 3D–F). It has been reported that LA BCF can be released from Piers’ photo LA generator (PhLAG)^[^ ^64^ ^]^ upon photo irradiation in the presence of a Lewis base.^[^ ^40^, ^41^, ^44^, ^64^ ^]^ The mixture of Si‐TMR and PhLAG with 1:1 ratio in toluene was colorless before light irradiation, and no reaction was observed. When irradiation at 254 nm for 1 min, the solution changed to a light green color, attributed to the solution of Si‐TMR‐BCF adduct **2**. After 6 min of irradiation, the color of the solution was further deepened to green. Meanwhile, the tracked UV‐Vis measurement showed a significant increase around 650 nm (Figure 3E). Similarly, addition of toluene to the mixture of spirocyclic TMR and PhLAG with a molar ratio of 1:1 led to a light pink solution. The absorption maximum around 550 nm appeared, which is attributed to the TMR‐BCF adduct **4**. The absorption did not show obvious variation within half an hour. However, upon irradiation at 254 nm, the color gradually darkens. Tracked UV‐Vis measurement showed a significant elevation around 550 nm in 6 min, and the absorption of spirocyclic TMR (305 nm) disappeared (Figure 3F). The color changes only when the irradiation starts and stops when the irradiation is shut off (Figure 3E,F). Here, spirolactone's nucleophilic CO_2_ moiety drives photolytic BCF release from PhLAG, initiating cascade coordination ring opening. Crucially, the system exhibits real‐time photoresponse—activation strictly requires UV exposure and halts immediately upon irradiation cessation, enabling spatiotemporal control.

## 3 Conclusion

We have demonstrated the first noninvasive ring opening of classical spirolactone rhodamines (Si‐TMR/TMR) via controlled LA complexation, achieving critical advancements over conventional protonation approaches in spectroscopic superiority, dynamic precision, and mechanistic breakthrough. Aluminum/boron/silicon‐coordinated derivatives (**3**–**5**) exhibit near‐unity quantum yields (Φ = 91.69–94.87%). Integration with Piers’ photoacid generator enables spatiotemporal control of fluorescence through UV‐induced BCF release. The Al/B/Si ← O coordination suppressed TICT extended excited‐state lifetimes to 6.49 ns. Moreover, the LA coordination‐induced electronic delocalization mechanism preserves molecular integrity while amplifying radiative decay—a critical advantage over destructive π‐extension strategies. This work paves a way towards the exploitation of rhodamine materials with outstanding fluorescent performance, such as spatiotemporally controllable fluorescent dyes. Also, these dyes may have potential applications in the fields of laser dyes, chemical sensors, organic light‐emitting diodes (OLEDs), and so on.

## Supporting Information

The authors have cited additional references within the Supporting Information.^[^ ^49^, ^50^, ^64^ ^]^

## Conflict of Interest

The authors declare no conflict of interest.

## Supporting information

Supplementary Material

## Data Availability

The data that support the findings of this study are available from the corresponding author upon reasonable request.
