# Supplementary material for: Lewis Acid‐Triggered Spatiotemporally Controllable Ring Opening in a Classic Rhodamine Featuring Φ = 95% Emission
Source: Small Sci. 2026 Jan 22;6(2):e202500522. doi: 10.1002/smsc.202500522 (PMC12915215; doi:10.1002/smsc.202500522)
Supplement: Supplementary file 1 — Supplementary Material [file SMSC-6-e202500522-s001.pdf]

Supporting Information

Lewis Acid-Trigged Spatiotemporally Controllable Ring-Opening in a Classic Rhodamine Featuring Φ = 95% Emission

Quanchun Sun,[a],[c] Liancheng He,[a],[c] Tao Wang,[a],[c] Haiyan Cui*[b], and Xinping Wang*[a],[c]

[a]State Key Laboratory of Coordination Chemistry, School of Chemistry and Chemical Engineering, Collaborative Innovation Center of Advanced Microstructures, Nanjing University.
[b]College of Sciences, Nanjing Agricultural University, Nanjing 210095, China.
[c]State Key Laboratory of Organometallic Chemistry, Shanghai Institute of Organic Chemistry, Chinese Academy of Science, Shanghai 200032, China.

Table of Contents

General methods..........2

Crystal data..........5

Selected bond length parameters of 1-5..........7

Mechanism study..........9

UV-vis spectra of Si-TMR and TMR..........10

Fluorescence lifetime (τ) of 1-5...........14

Reversibility investigation..........18

NMR spectra of 1-5..........20

References..........27

General methods

All experiments involving air-sensitive compounds are performed in the glove box or using the Schlenk technique in N_2 atmospheres. Similarly, if the solvent used in the experiment is water-free and oxygen-free, the solvent is dehydrated and deoxygenated by standard methods, and the solvent is treated in the present. The NMR spectra were collected on Brucker DRX NMR spectrometer, and the chemical shift values were calibrated using the residual solvent peak of the deuterium used in the test as the internal standard. The UV-Vis absorption spectra were recorded by the Lambda 750 spectrophotometer. Fluorescence data were measured at room temperature with a FLS 1000 spectrometer. The determined crystal structure data were collected and reduced at Brucker CMOS X-ray single crystal diffractometer. Element analysis were performed on an Elementar Vario EL III instrument at Shanghai Institute of Organic Chemistry, Chinese Academy of Sciences. EPR spectra were obtained using a Brucker EMX plus-6/1 X-band variable-temperature apparatus. The precursor compounds TMR and Si-TMR were synthesized according to the methods reported in the literatures[46], [47], all of which were dried before use. PhLAG was synthesized according to the method reported in the literature[61].

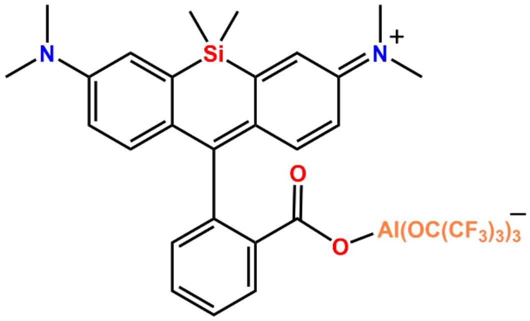

Synthesis of 1: Toluene (~20 mL) was added into the mixture of Si-TMR (0.043 g, 0.1 mmol) and $Al(OC(CF_3)_3)_3$ (FC_6H_5) (0.11 mmol，~0.25 mL) in a 100 mL Schlenk flask in the glove box. The reaction mixture was stirred overnight at room temperature, then the resultant dark blue solution was filtered. The filtrate was condensed and stored at -20 °C for two days to obtain green flaky crystals. Yield: 21 mg (crystal yield); ^{1}H NMR (400 MHz, $CDCl_3$): δ = 7.97 (d, 1H), 7.52 (t, 1H); 7.64 (t, 1H); 7.54 (t, 2H); 7.31 (d, 1H); 6.97 (s, 2H); 6.77 (d, 2H); 6.57 (d, 2H); 2.96 (s, 12H); 0.63 (s, 3H); 0.61 (s, 3H); ^{19}F NMR (376 MHz,

$CDCl_3$): δ = -75.41; ^{29}Si NMR (79 MHz, $CDCl_3$): δ = -113.93. Elemental analysis for $C_{38}H_{28}AlF_{27}N_2O_5Si$ (%): Cacld: C 39.32, H 2.43, N 2.41; Found: C 37.52, H 2.19, N 2.16. The melting points is above 300 °C.

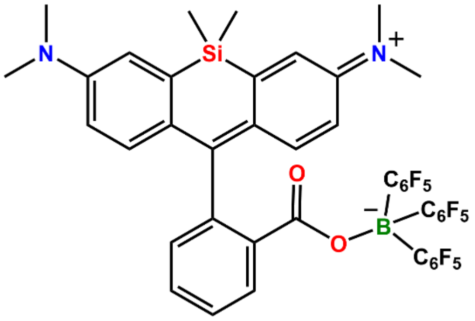

Synthesis of 2: Toluene (~20 mL) was added into the mixture of Si-TMR (0.043 g, 0.1 mmol) and $B(C_6F_5)_3$ (0.1 mmol，0.0512 g) in a 100 mL Schlenk flask in the glove box. The reaction mixture was stirred overnight at room temperature, then the resultant dark blue solution was filtered. The filtrate was condensed mildly and stored at room temperature for 10 minutes to obtain blue flaky crystals. Yield: 19.5mg (crystal yield); ^1H NMR (400 MHz, $CDCl_3$): δ = 8.30 (d, 1H), 7.52 (t, 1H); 7.42 (t, 1H); 7.03 (d, 2H); 6.96 (d, 1H); 6.84 (s, 2H); 6.45 (d, 2H); 3.20 (s, 12H); 0.44 (s, 3H); 0.22 (s, 3H); ^{11}B NMR (128 MHz, CD_2Cl_2): δ = -5.36; ^{19}F NMR (376 MHz, CD_2Cl_2): δ = (-133.81, -163.07, -166.63); ^{29}Si NMR (79 MHz, $CDCl_3$): δ = -117.72. Elemental analysis for $C_{44}H_{28}F_{15}N_2O_2BSi$ (%): Cacld: C 56.18, H 3.00, N 2.97; Found: C 59.43, H 3.42, N 2.68. The melting points is above 300 °C.

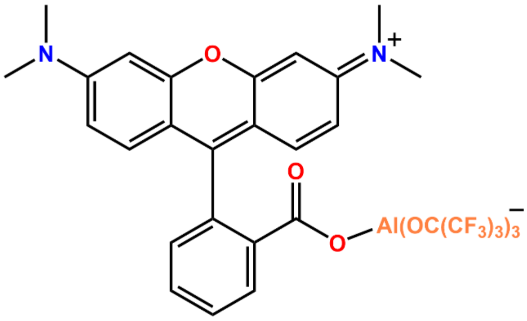

Synthesis of 3: Fluorobenzene (~50 mL) was added into the mixture of TMR (0.0387 g, 0.1 mmol) and $Al(OC(CF_3)_3)_3•C_6H_5F$ (0.11 mmol, ~0.25 mL) in a 100 mL Schlenk flask in the glove box. The reaction mixture was stirred overnight at room temperature, then the resultant bright red solution was filtered. The filtrate was condensed and stored at room temperature for 30 minutes to obtain amaranth massive crystals. Yield: 12.8 mg (crystal yield); ^1H NMR (400 MHz, CD_2Cl_2): δ = 8.24 (dd, 1H), 7.66 (m, 2H); 7.24 (s, 1H); 7.21 (t,

2H); 6.85 (dd, 2H); 6.78 (d, 2H); 3.25 (s, 12H); ^{19}F NMR (376.56 MHz, CD_2Cl_2): δ = -75.69; ^{27}Al NMR (104.28 MHz, CD_2Cl_2): δ = 39.10. Elemental analysis for $C_{36}H_{22}AlF_{27}N_2O_6$ (%): Cacld: C 38.65, H 1.98, N 2.5; Found: C 38.54, H 1.90, N 2.65. The melting points is above 300 °C.

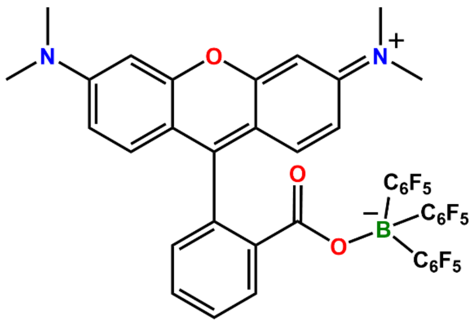

Synthesis of 4: Toluene (~50 mL) was added into the mixture of TMR (0.0387 g, 0.1 mmol) and $B(C_6F_5)_3$ (0.11 mmol, 0.0563 g) in a 100 mL Schlenk flask in the glove box. The reaction mixture was stirred overnight at room temperature, then the resultant bright red solution was filtered. The filtrate was condensed and stored at -20 °C for two days, and sequentially for about two weeks at room temperature to obtain red massive crystals. Yield: 11 mg (crystal yield); ^{1}H NMR (400 MHz, CD_2Cl_2): δ = 8.24 (d, 1H), 7.63 (t, 1H); 7.55 (t, 1H); 7.16 (d, 1H); 7.08 (d, 2H); 6.75 (dd, 2H); 6.59 (d, 2H); 3.16 (s, 12H); ^{11}B NMR (128 MHz, CD_2Cl_2): δ = -4.75; ^{19}F NMR (376 MHz, CD_2Cl_2): δ = (-134.44, -163.24, -167.04); Elemental analysis for $C_{42}H_{22}BF_{15}N_2O_3$ (%): Cacld: C 56.14, H 2.46, N 3.12; Found: C 57.13, H 2.94, N 2.74. The melting points is above 300 °C.

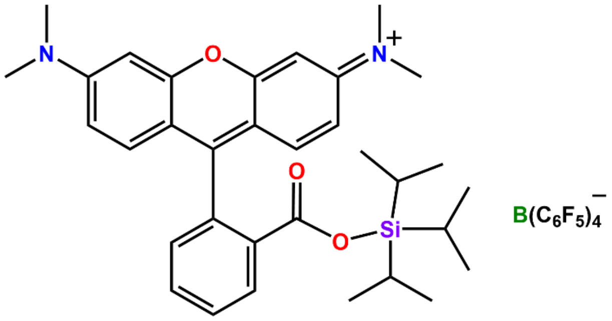

Synthesis of 5: Toluene (~50 mL) was added into the mixture of TMR (0.0387 g, 0.1 mmol) and $Si(CH(CH_3)_2)_3(B(C_6F_5)_4)$ (0.1 mmol, ~0.5 mL) in a 100 mL Schlenk flask in the glove box. The reaction mixture was stirred overnight at room temperature, then the resultant amaranth solution was filtered. The filtrate was evaporated to remove the solvent and then added to the diethyl ether solvent (~10 mL), stored at -20 °C for two days to obtain

red strip crystals. Yield: 10 mg (crystal yield); ^{1}H NMR (400 MHz, $CDCl_3$): δ = 8.20 (d, 1H), 7.67 (m, 2H); 7.19 (m, 1H); 7.03 (d, 1H); 6.91 (d, 1H); 6.7. (dd, 1H); 6.63 (m, 2H); 6.55 (d, 1H); 3.18 (s, 12H); 1.34 (s, 3H); 1.04 (s, 12H); 0.85 (s, 14H); ^{29}Si NMR (79 MHz, $CDCl_3$): δ = -114.07; ^{13}C NMR (101 MHz, $CDCl_3$): δ = 156.67, 156.55, 156.19, 131.63, 130.60, 130.15, 129.87, 129.49, 129.20, 112.96, 112.89, 112.78, 112.68, 95.49, 64.83, 58.51, 39.62, 39.53, 37.14, 30.21, 16.58, 14.23, 10.93. Elemental analysis for $C_{57}H_{43}BF_{20}N_2O_3$ (%): Cacld: C 55.98, H 3.54, N 2.29; Found: C 54.91, H 3.33, N 2.28. The melting points is above 300 °C.

Crystal data

Table S1. Crystallographic data of **1** and **2**.

	**1**	**2**
CCDC No.	2422754	2422758
Formula	$C_{38}H_{28}AlF_{27}N_2O_5Si$	$C_{44}H_{28}BF_{15}N_2O_2Si$
*M*r[g/mol]	1160.69	940.58
Crystal system	Monoclinic	Triclinic
Space group	$P2_1/c$	*P*1
Z	4	4
Temp.(K)	123	193
μ (mm^{-1})	1.17	0.66
a (Å)	16.7156(12)	13.3140(3)
b (Å)	16.1580(12)	18.3920(4)
c (Å)	20.1547(15)	24.9150(6)
α (°)	90.000	100.361(9)
β (°)	105.795(3)	93.573(9)
γ (°)	90.000	95.681(7)
V [Å^3]	5238.1(7)	5952.0(2)
R_1 ($I>2\sigma(I)$)	0.079	0.117
wR_2 (all data)	0.253	0.388

$R_1 = \Sigma||F_o| - |F_c||/\Sigma F_o|$， $wR_2 = [\Sigma w(F_o^2 - F_c^2)^2/\Sigma w(F_o^2)^2]^{1/2}$

Table S2. Crystallographic data of **3-5**.

	**3**	**4**	**5**
CCDC No.	2422755	2422756	2422757
Formula	$C_{36}H_{22}AlF_{27}N_2O_6$	$C_{42}H_{22}BF_{15}N_2O_3$	$C_{57}H_{43}BN_2O_3SiF_{20}$
M_r [g/mol]	1118.54	898.42	1222.83
Crystal system	Triclinic	Triclinic	Triclinic
Space group	$P1$	$P1$	$P1$
Z	2	2	2
Temp.(K)	193	193	193
μ (mm^{-1})	0.17	0.16	0.94
a (Å)	13.0798(4)	10.8323(7)	11.3990(16)
b (Å)	15.0706(5)	10.8724(7)	15.2820(2)
c (Å)	15.4061(5)	17.8548(11)	17.0320(2)
α (°)	67.320(1)	80.2930(2)	64.5120(4)
β (°)	86.0570(1)	81.1680(2)	83.8730(5)
γ (°)	73.5070(1)	62.1140(2)	86.2120(5)
V [Å^3]	2683.8900(15)	1825.3000(2)	2662.3000(6)
R_1 (I>2σ(I))	0.0796	0.0559	0.0515
wR_2 (all data)	0.2471	0.1494	0.160

$R_1 = \Sigma||F_o| - |F_c||/\Sigma F_o|$, $wR_2 = [\Sigma w(F_o^2 - F_c^2)^2/\Sigma w(F_o^2)^2]^{1/2}$

Selected bond length parameters of 1-5

Table S3. Selected bond length parameters of **1** and **2** (Cal: theoretical calculation results, Exp: crystal experiment results) (Å).

	1		2	
Bond	Exp	Cal	Exp	Cal
C1-O1	1.203(6)	1.233	1.230(10)	1.204
C1-O2	1.326(6)	1.302	1.329(9)	1.302
C2-C3	1.424(7)	1.441	1.403(9)	1.436
C2-C15	1.510(4)	1.503	1.518(10)	1.503
C3-C4	1.433(7)	1.422	1.448(10)	1.425
C4-C5	1.355(8)	1.377	1.396(10)	1.374
C5-C6	1.419(4)	1.419	1.452(10)	1.422
C6-C7	1.415(4)	1.424	1.431(10)	1.426
C7-C8	1.376(4)	1.385	1.375(10)	1.383
C8-C3	1.437(4)	1.432	1.435(9)	1.433
C9-C10	1.365(4)	1.383	1.407(10)	1.385
C9-C14	1.430(4)	1.432	1.400(9)	1.432
C10-C11	1.431(5)	1.425	1.419(10)	1.424
C11-C12	1.405(5)	1.421	1.412(10)	1.419
C12-C13	1.355(5)	1.374	1.346(10)	1.377
C13-C14	1.427(5)	1.423	1.433(9)	1.422
C14-C2	1.420(4)	1.437	1.405(10)	1.441
N1-C11	1.351(13)	1.364	1.350(10)	1.365
N2-C6	1.347(4)	1.366	1.333(9)	1.360

Table S4. Selected bond length parameters of **3**, **4** and **5** (Cal: theoretical calculation results, Exp: crystal experiment results) (Å).

	**3**		**4**		**5**	
Bond	Exp	Cal	Exp	Cal	Exp	Cal
C1-O1	1.209(4)	1.228	1.219(3)	1.229	1.198(4)	1.223
C1-O2	1.307(4)	1.301	1.311(4)	1.303	1.322(3)	1.331
C2-C3	1.392(5)	1.416	1.399(4)	1.412	1.394(4)	1.411
C2-C15	1.496(5)	1.493	1.489(4)	1.493	1.494(4)	1.496
C3-C4	1.429(5)	1.421	1.423(4)	1.423	1.409(4)	1.424
C4-C5	1.356(6)	1.370	1.360(4)	1.369	1.354(4)	1.368
C5-C6	1.432(5)	1.433	1.422(4)	1.436	1.437(4)	1.437
C6-C7	1.417(5)	1.412	1.407(4)	1.415	1.393(4)	1.416
C7-C8	1.369(5)	1.384	1.370(4)	1.382	1.372(4)	1.381
C8-C3	1.422(5)	1.421	1.410(4)	1.422	1.421(4)	1.424
C9-C10	1.380(5)	1.383	1.362(4)	1.384	1.366(4)	1.381
C9-C14	1.421(5)	1.421	1.417(4)	1.420	1.417(4)	1.423
C10-C11	1.403(5)	1.414	1.408(4)	1.413	1.405(4)	1.415
C11-C12	1.411(6)	1.434	1.420(4)	1.433	1.432(4)	1.437
C12-C13	1.364(6)	1.368	1.358(4)	1.371	1.357(5)	1.369
C13-C14	1.420(5)	1.422	1.423(4)	1.421	1.419(4)	1.424
C14-C2	1.404(5)	1.414	1.400(4)	1.416	1.405(4)	1.411
N1-C11	1.358(5)	1.351	1.348(4)	1.365	1.351(4)	1.358
N2-C6	1.336(5)	1.365	1.352(4)	1.360	1.350(4)	1.358

Mechanism study

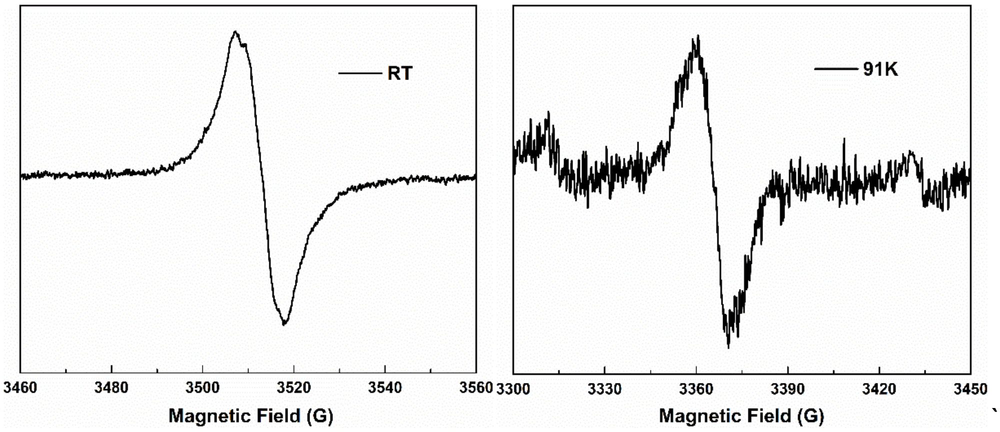

Figure S1. EPR spectra of TMR+BCF in toluene in suit.

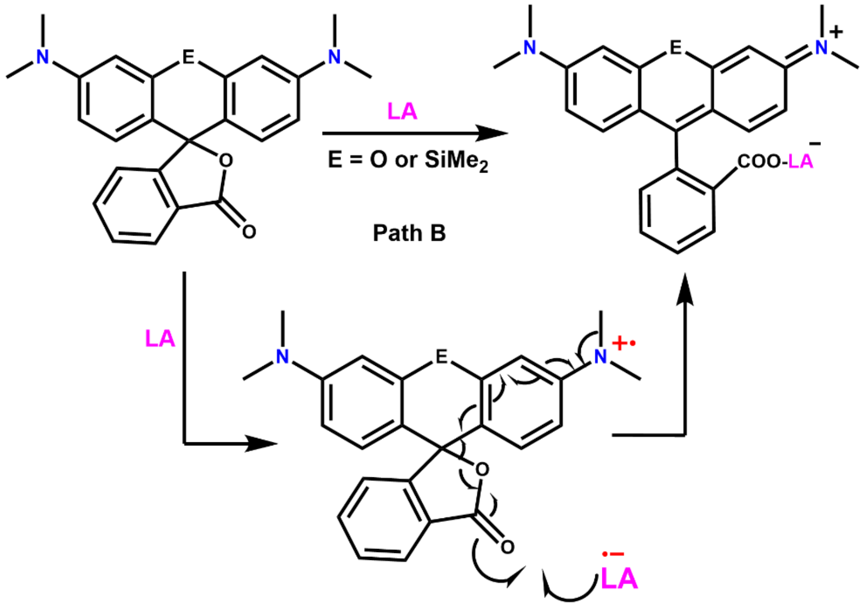

Figure S2. One proposed mechanism for the open-ring process of rhodamine spirolactones with Lewis acids.

UV-vis spectra of Si-TMR and TMR

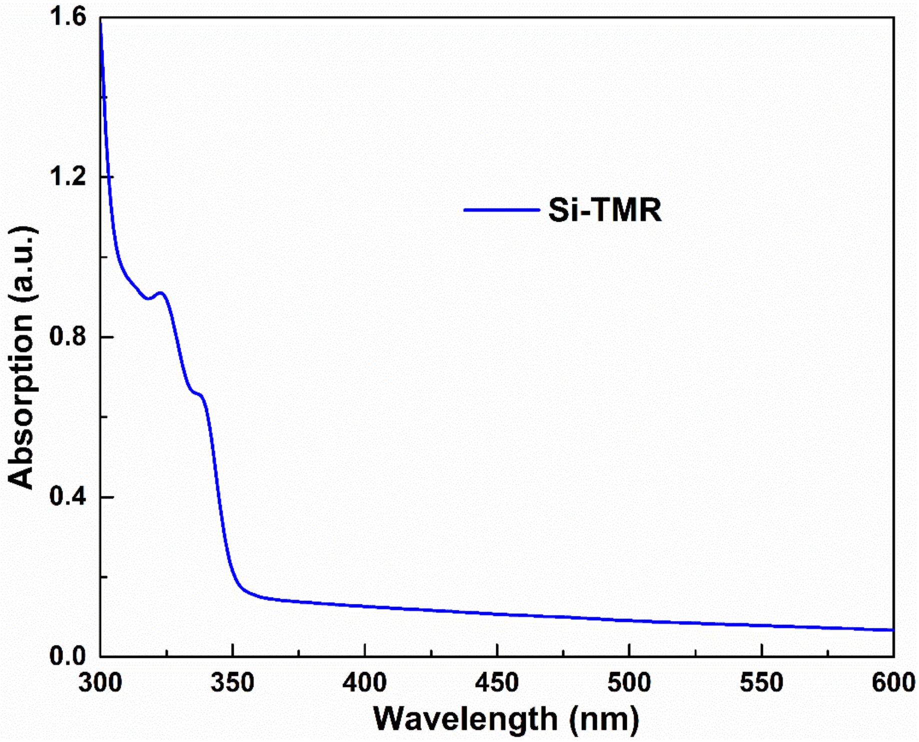

Figure S3. UV-Vis spectrum of Si-TMR in toluene.

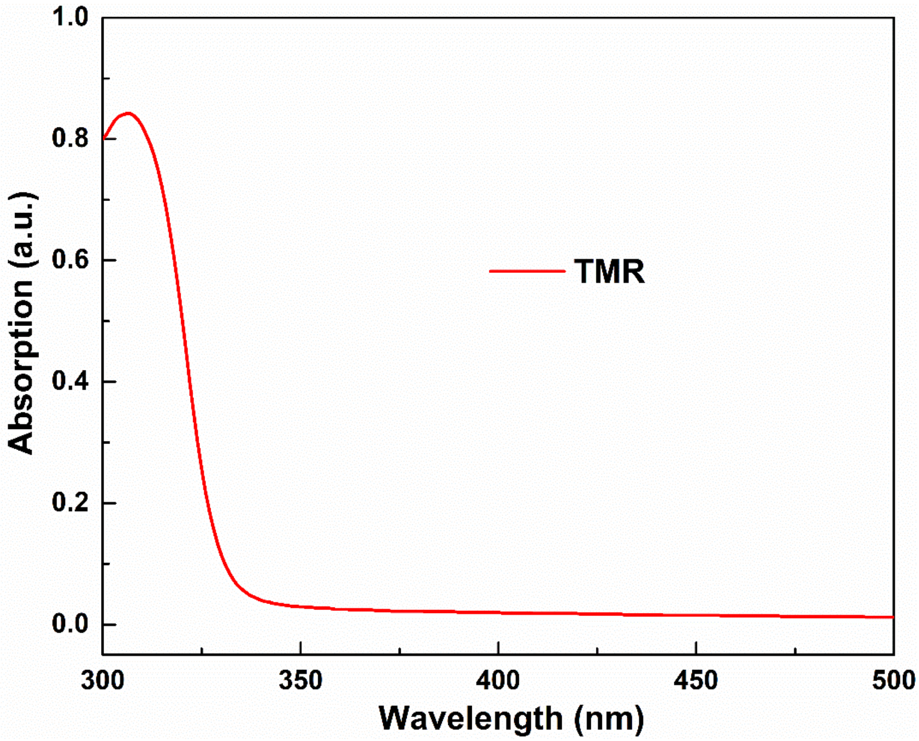

Figure S4. UV-Vis spectrum of TMR in toluene.

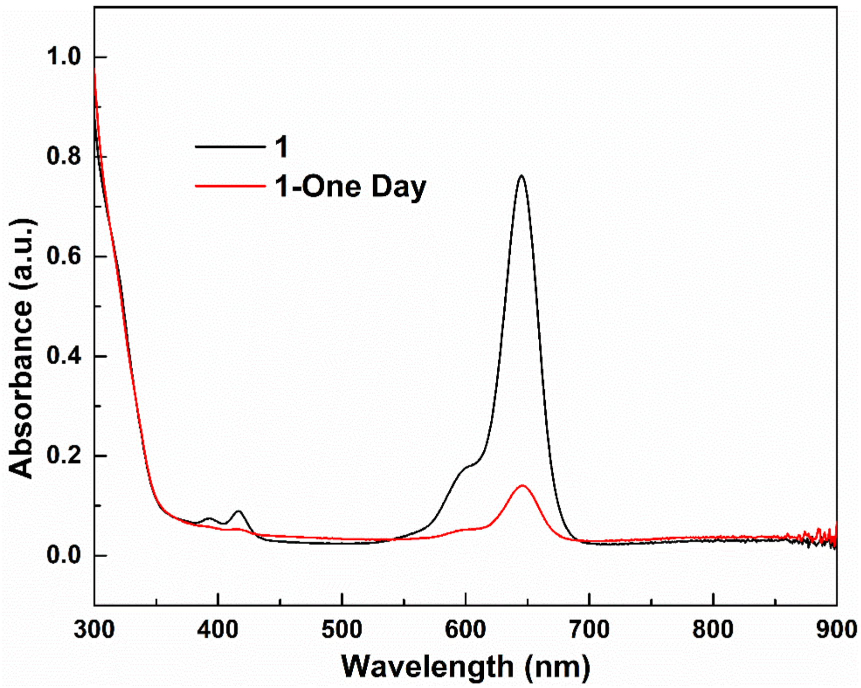

Figure S5. Evolution of the UV-Vis spectrum of compound **1** in toluene over one day in the air.

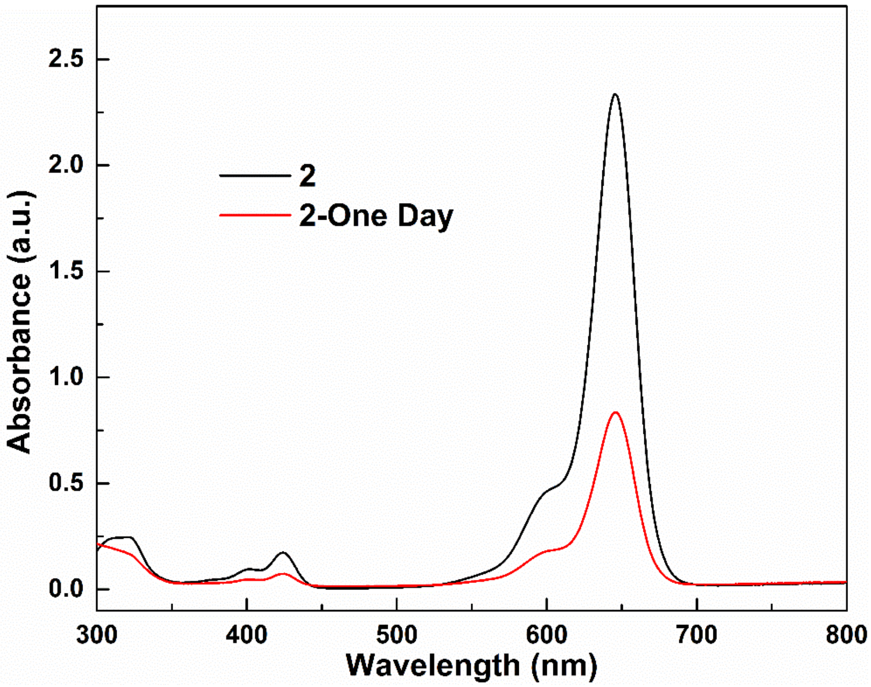

Figure S6. Evolution of the UV-Vis spectrum of compound **2** in toluene over one day in the air.

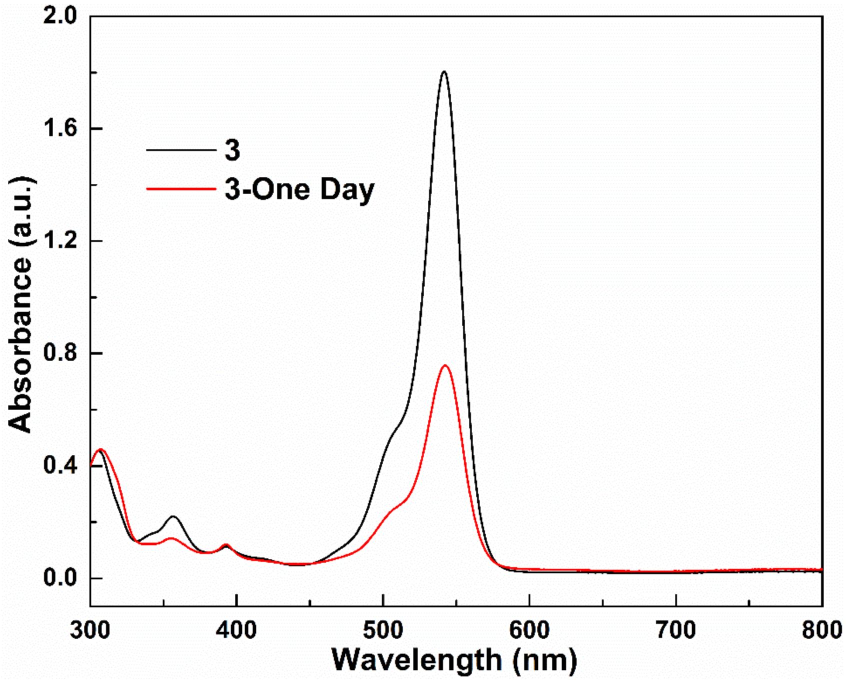

Figure S7. Evolution of the UV-Vis spectrum of compound **3** in toluene over one day in the air.

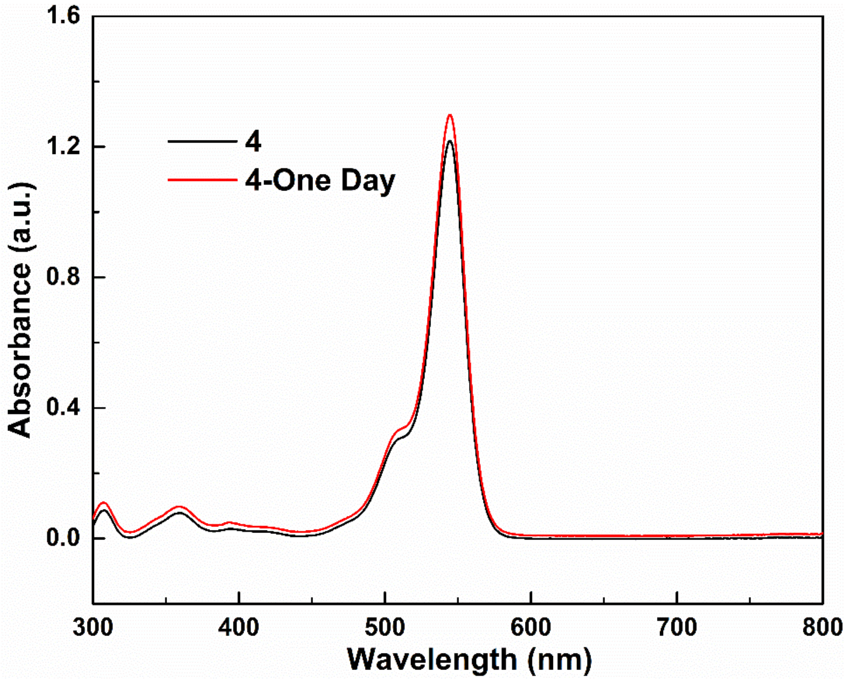

Figure S8. Evolution of the UV-Vis spectrum of compound **4** in toluene over one day in the air.

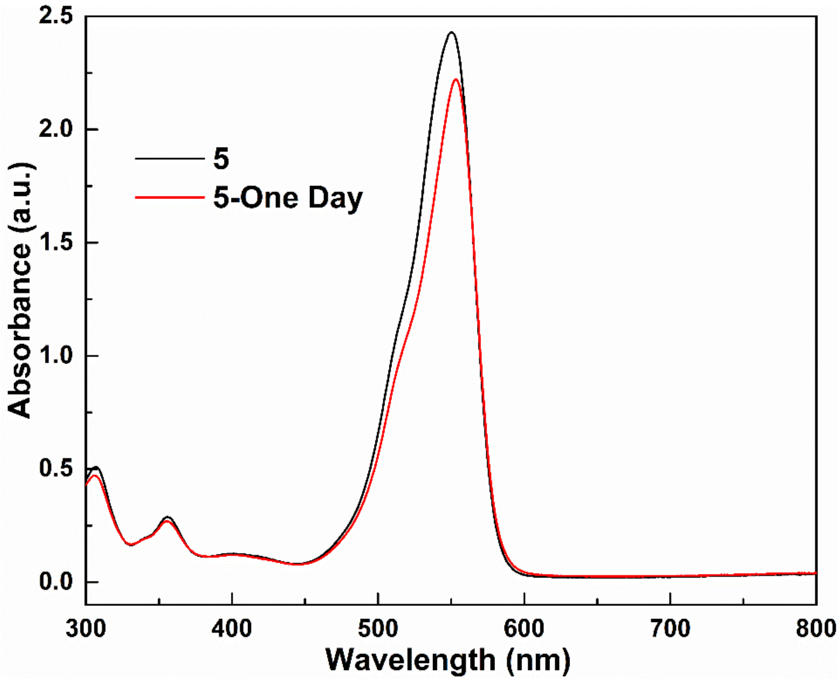

Figure S9. Evolution of the UV-Vis spectrum of compound **5** in toluene over one day in the air.

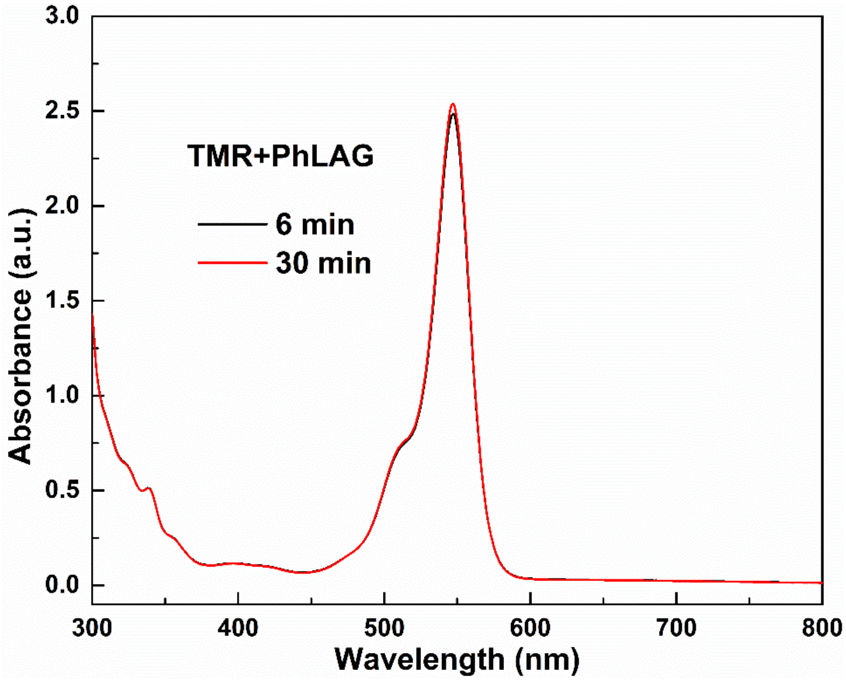

Figure S10. UV-Vis spectrum of compound **TMR+PhLAG** in toluene at different UV light times.

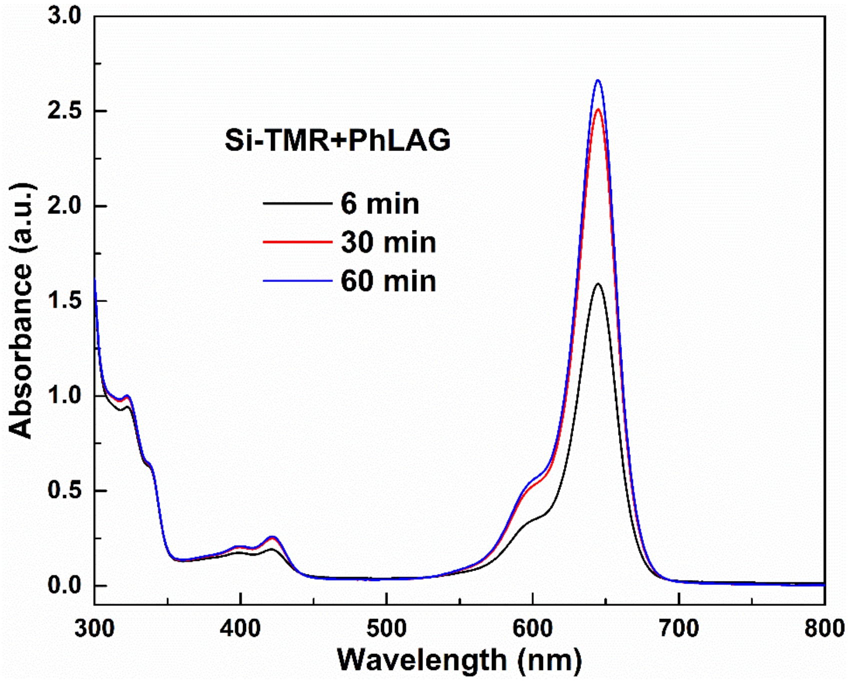

Figure S11. UV-Vis spectrum of compound **Si-TMR+PhLAG** in toluene at different UV light times.

Fluorescence lifetime (τ) of 1-5.

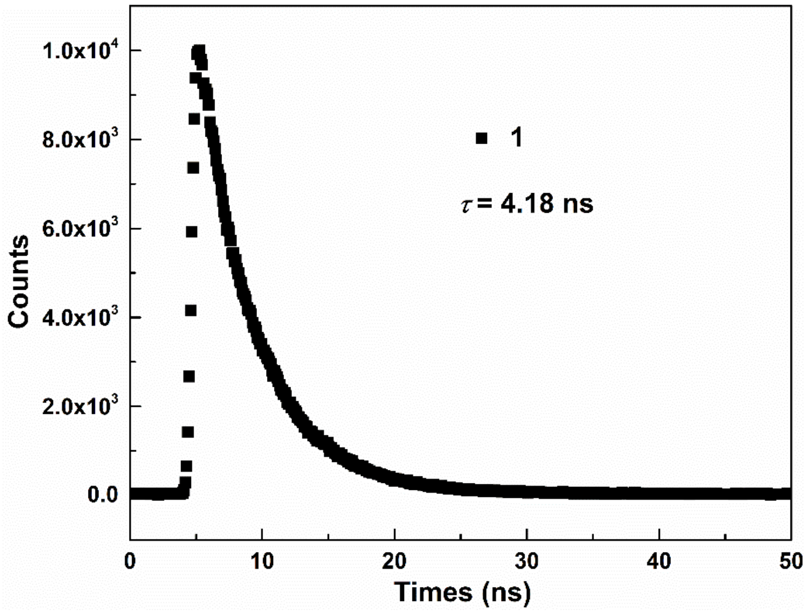

Figure S12. Fluorescence lifetime of **1** in toluene.

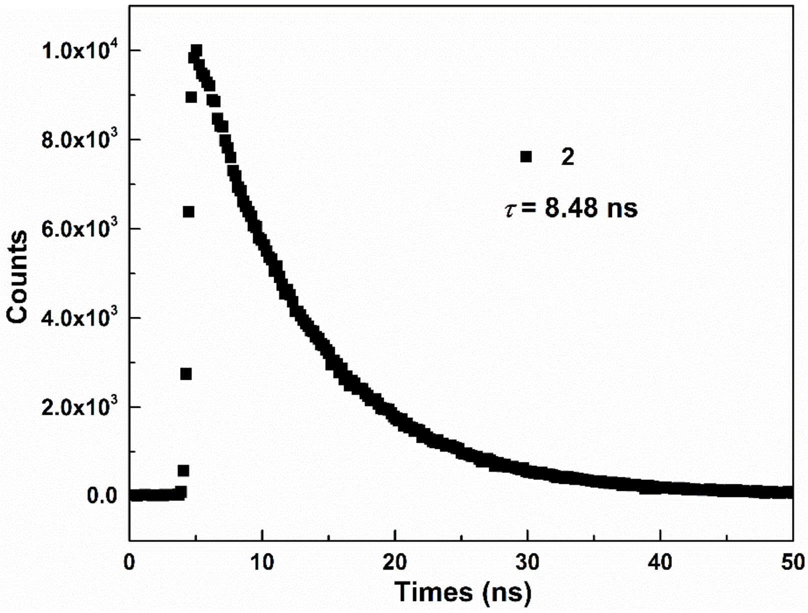

Figure S13. Fluorescence lifetime of **2** in toluene.

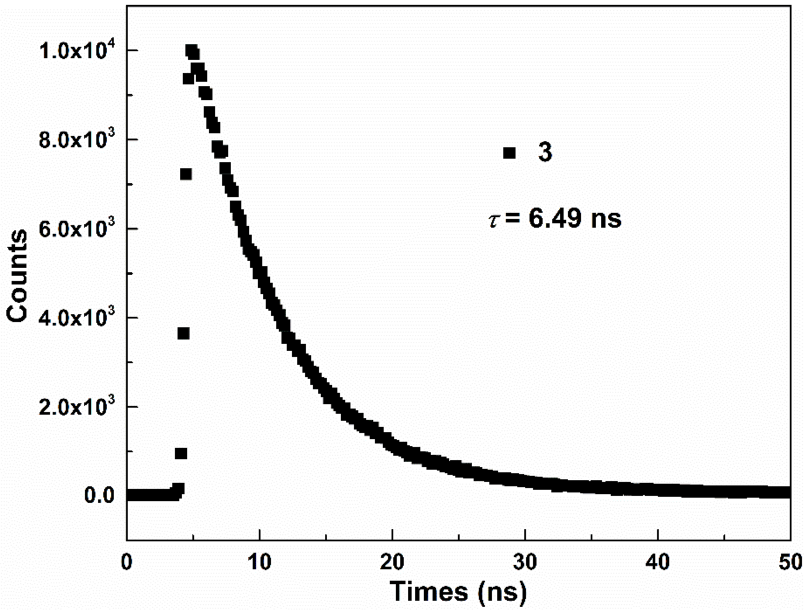

Figure S14. Fluorescence lifetime of **3** in in toluene.

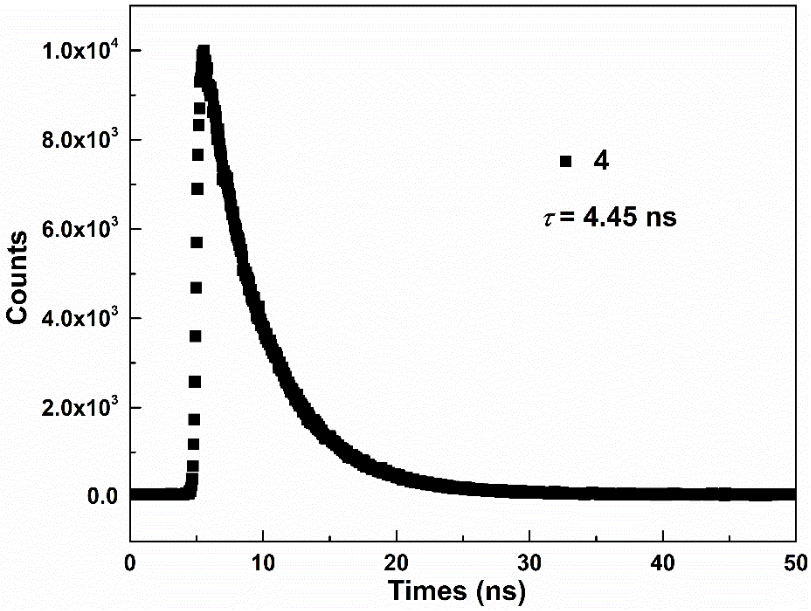

Figure S15. Fluorescence lifetime of **4** in toluene.

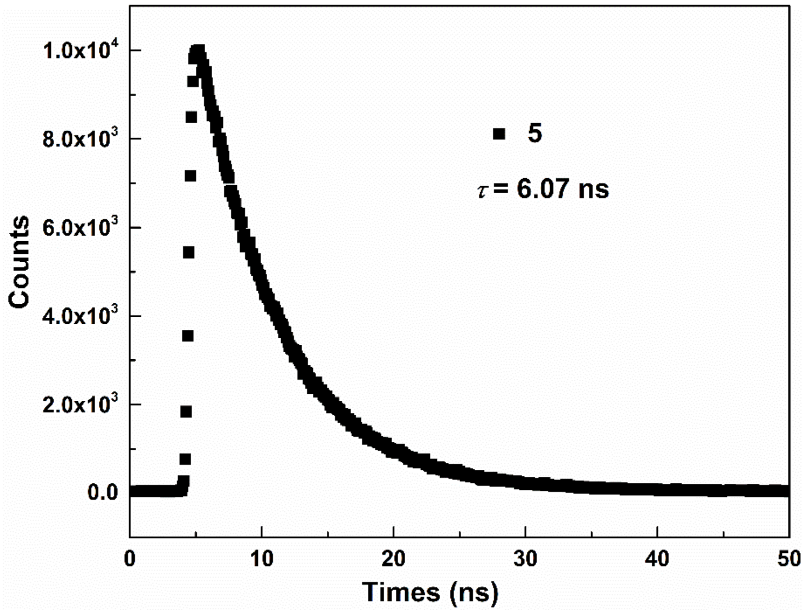

Figure S16. Fluorescence lifetime of **5** in toluene.

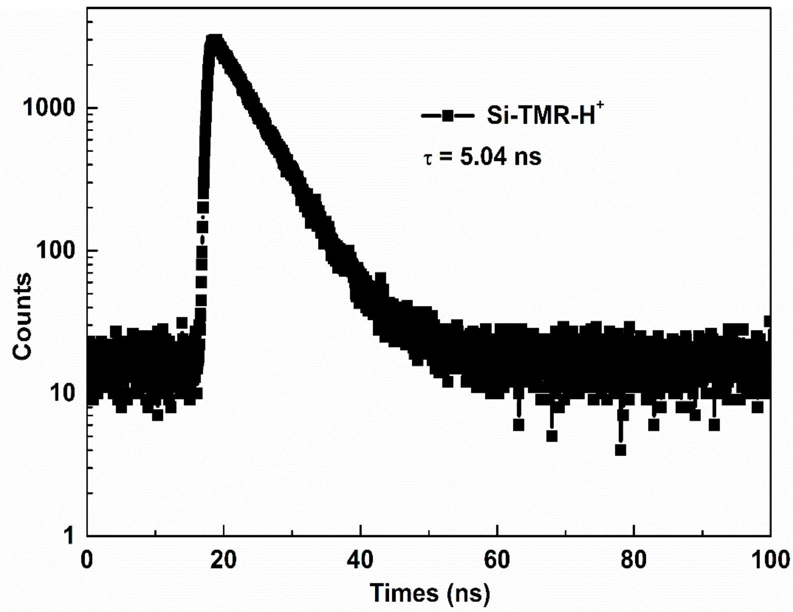

Figure S17. Fluorescence lifetime of Si-TMR-H$^+$ [in 0.1%(v/v) TFA-EtOH].

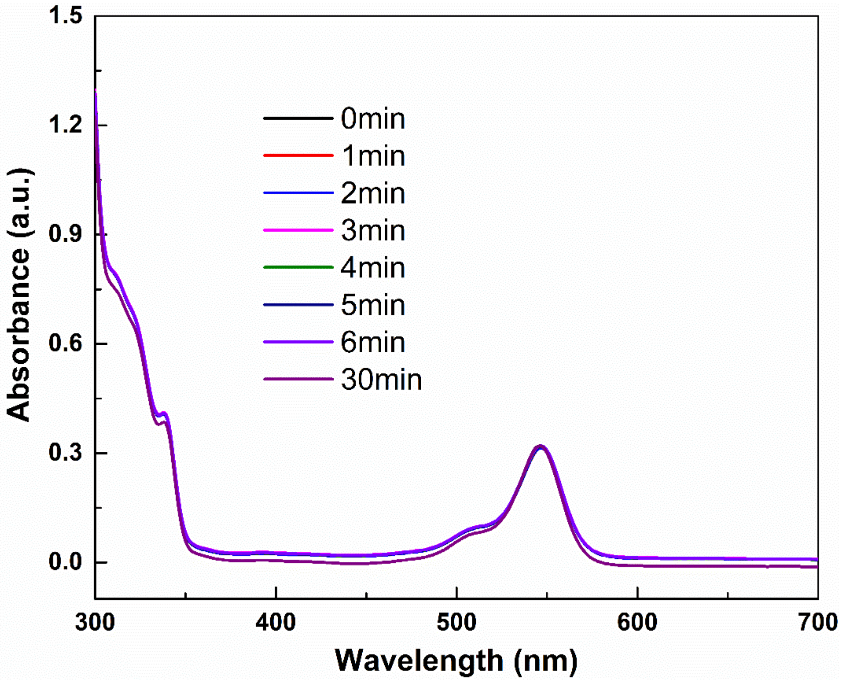

Figure S18. The UV-Vis spectra of TMR with PhLAG at different times under natural light.

Reversibility investigation

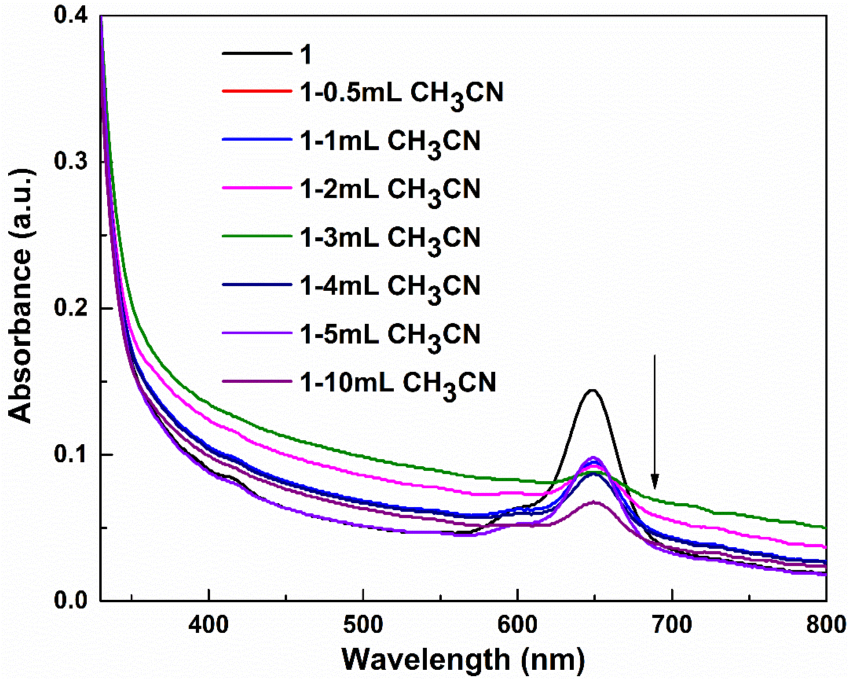

Figure S19. The UV-Vis spectrum of **1** under the addition of different amounts of acetonitrile.

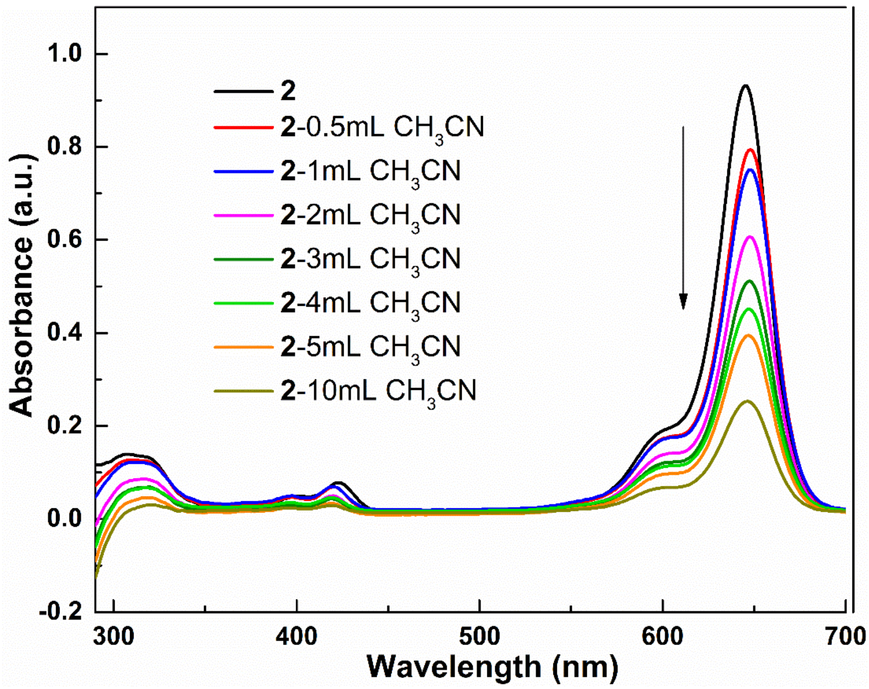

Figure S20. The UV-Vis spectrum of **2** under the addition of different amounts of acetonitrile.

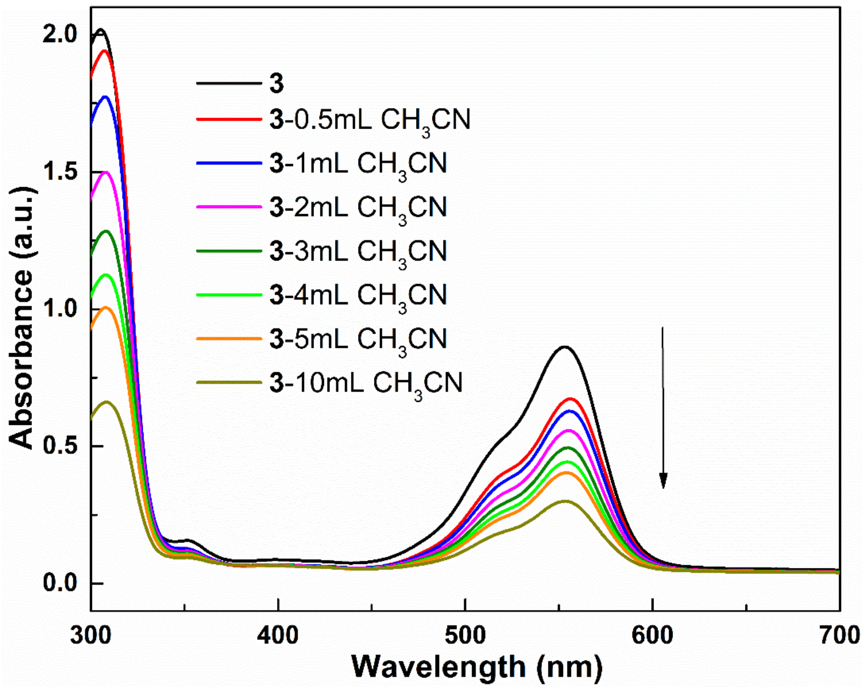

Figure S21. The UV-Vis spectrum of **3** under the addition of different amounts of acetonitrile.

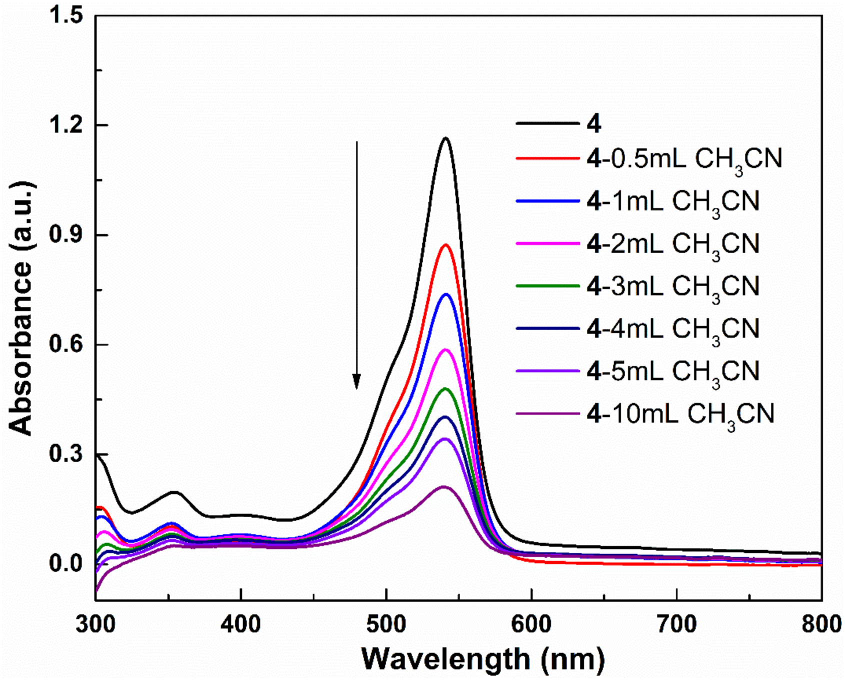

Figure S22. The UV-Vis spectrum of **4** under the addition of different amounts of acetonitrile.

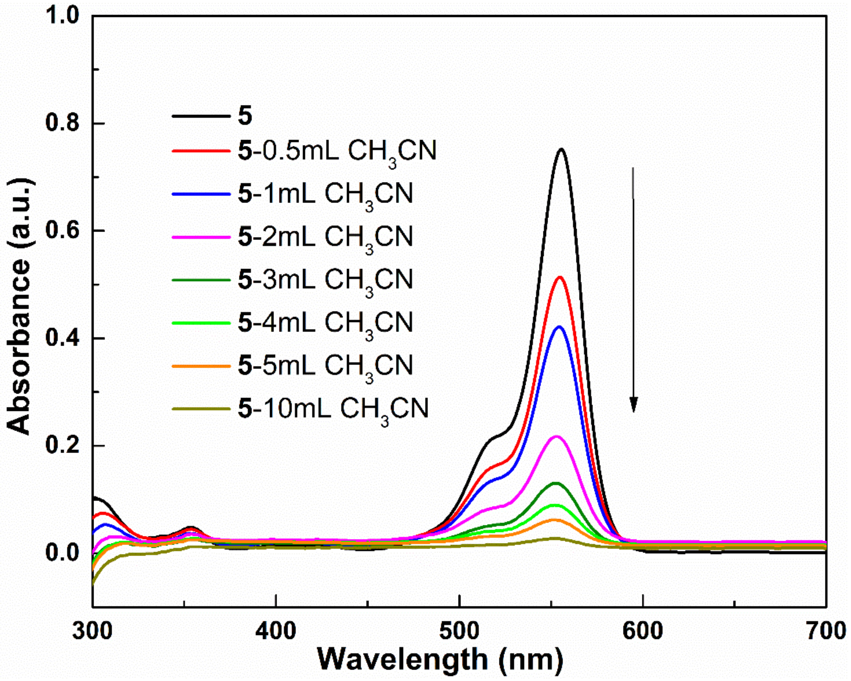

Figure S23. The UV-Vis spectrum of **5** under the addition of different amounts of acetonitrile.

NMR spectra of 1-5

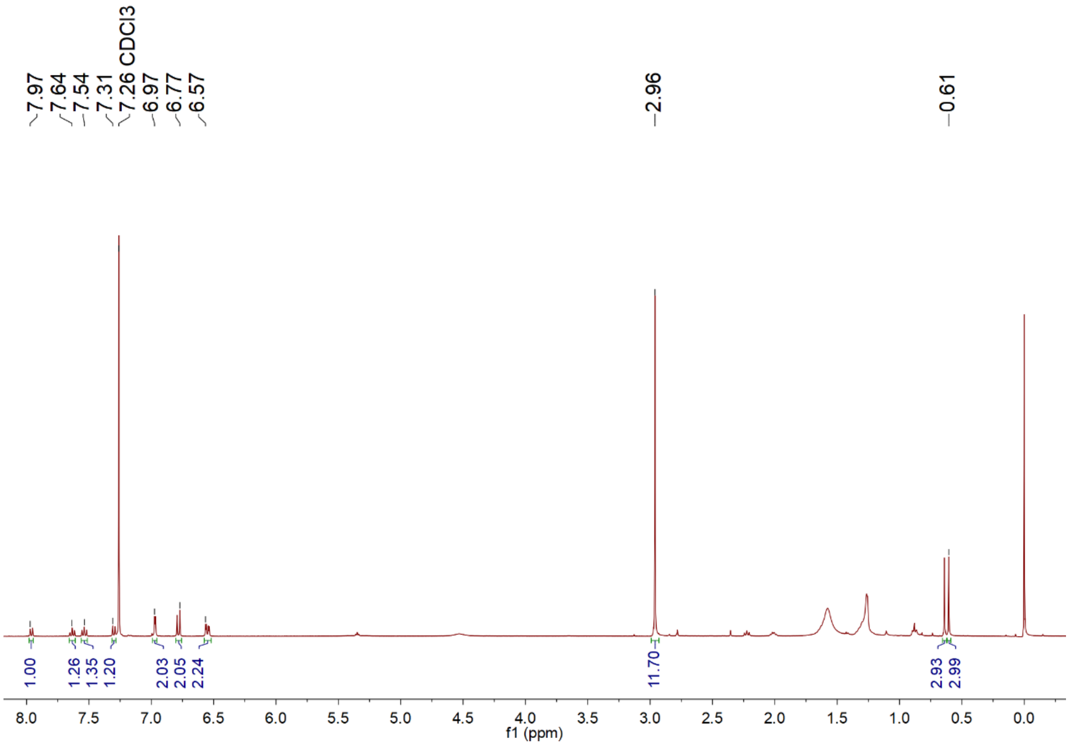

Figure S24. ^{1}H NMR spectrum of **1** in $CDCl_3$.

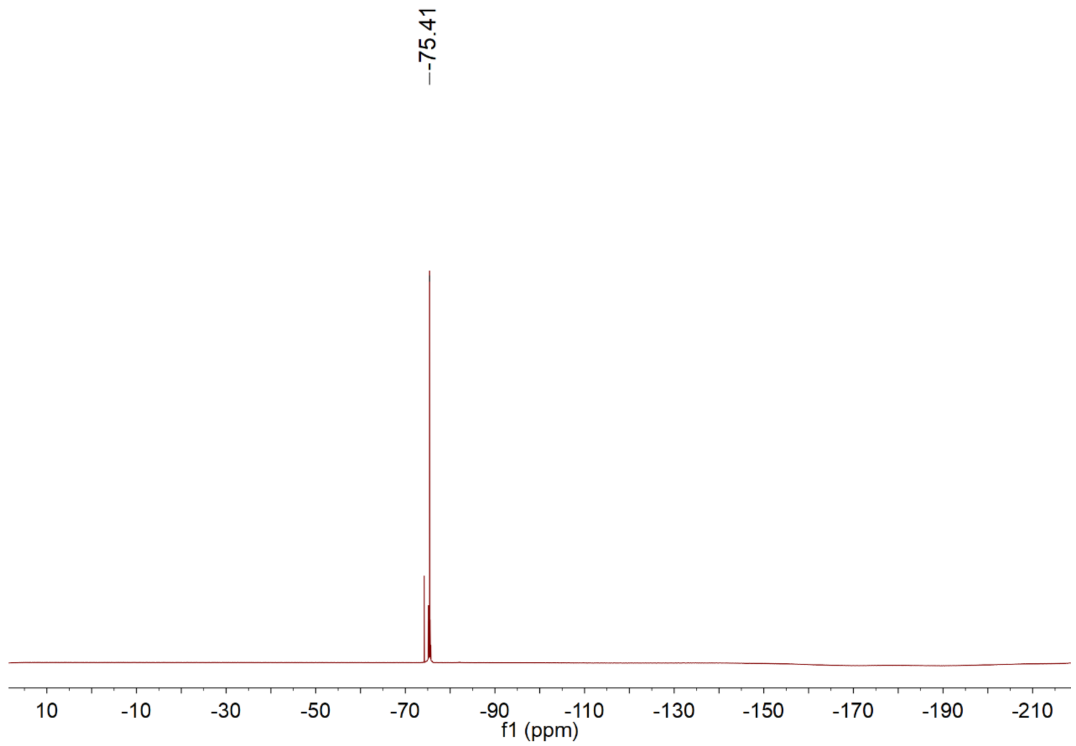

Figure S25. ^{19}F NMR spectrum of **1** in $CDCl_3$.

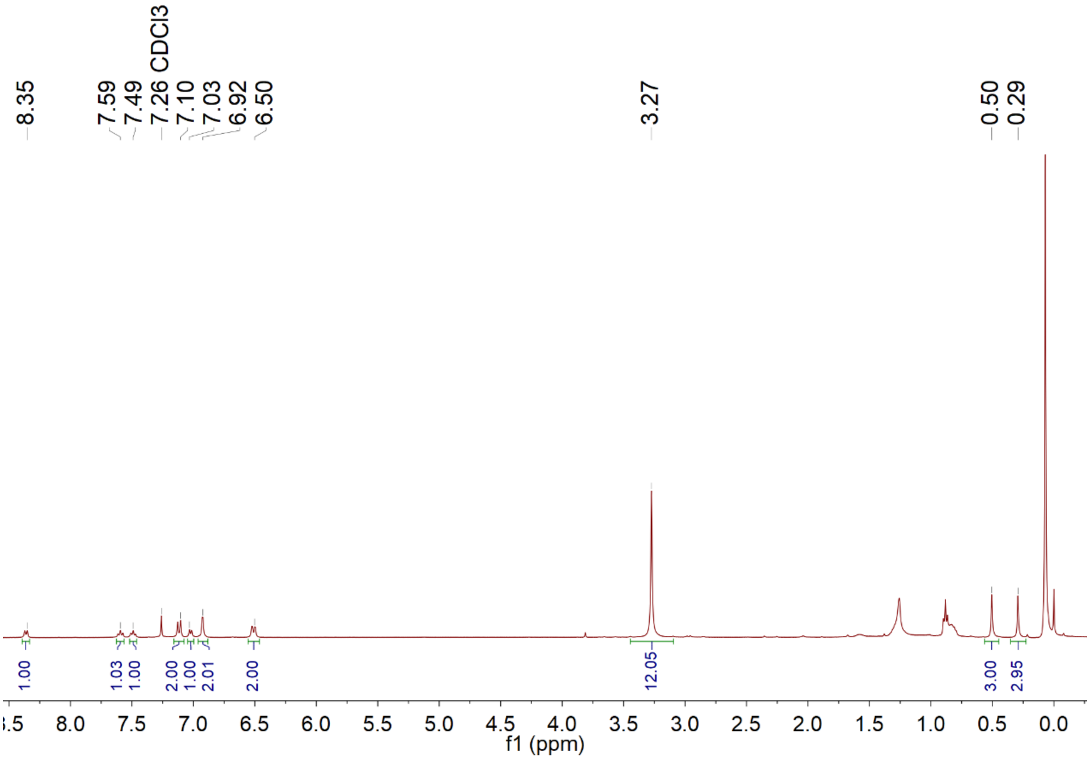

Figure S26. ^{1}H NMR spectrum of **2** in $CDCl_3$.

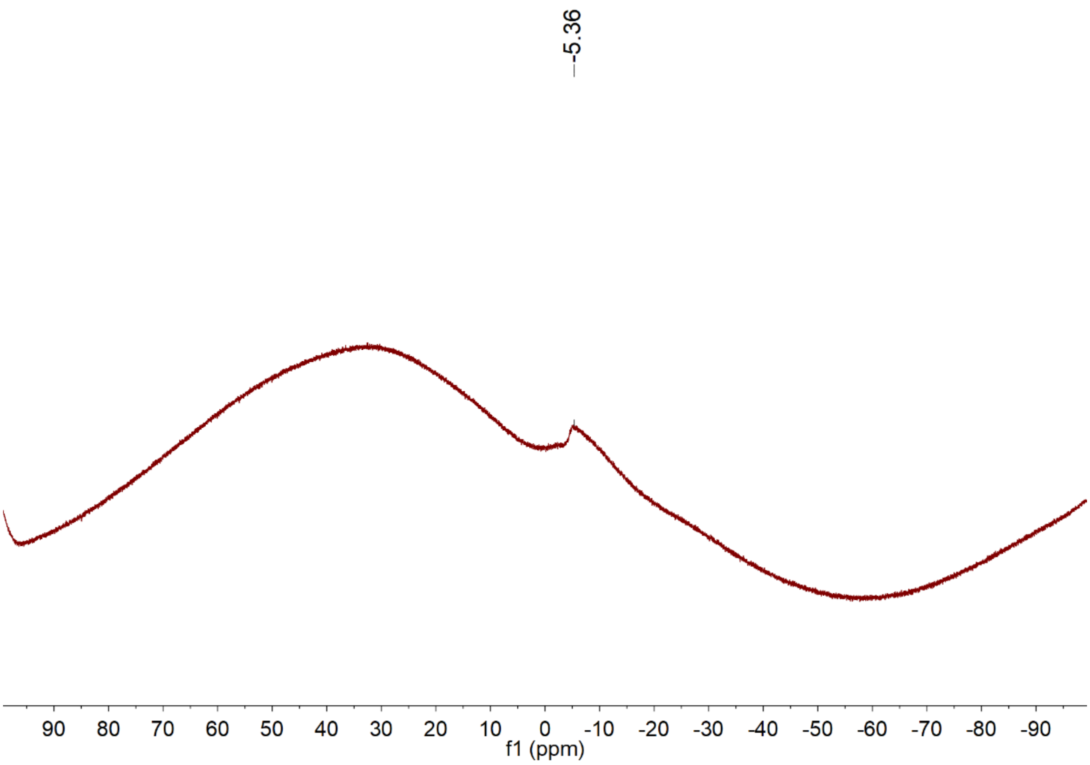

Figure S27. ^{11}B NMR spectrum of **2** in $CDCl_3$.

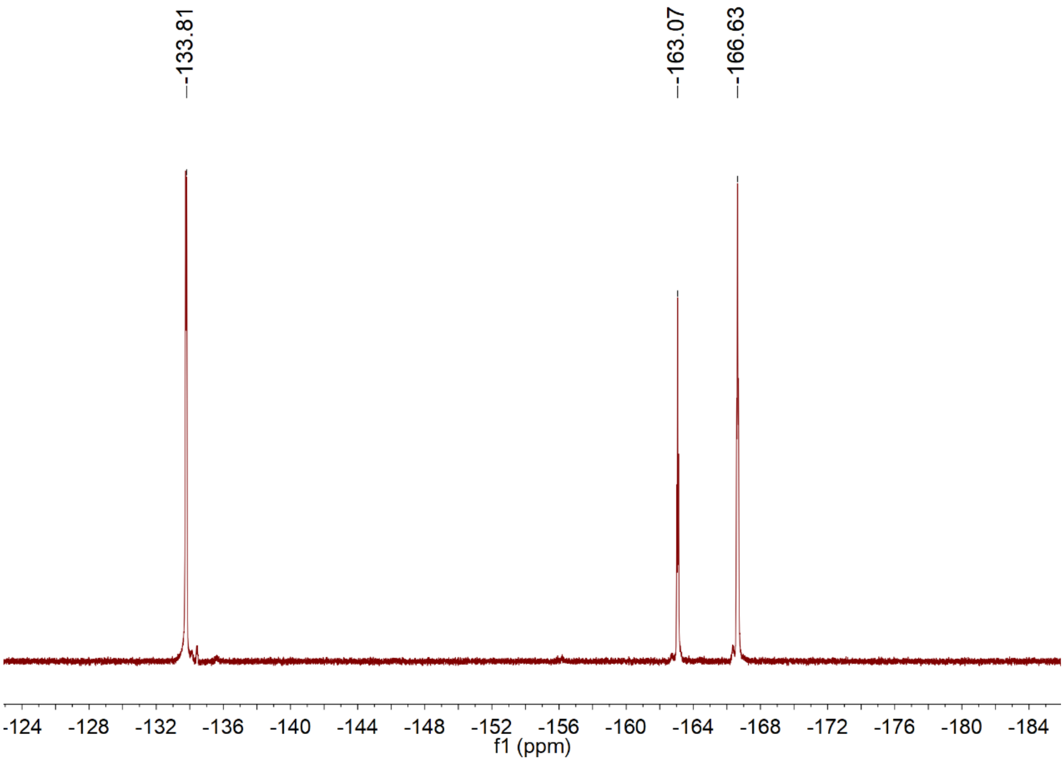

Figure S28. ^{19}F NMR spectrum of **2** in $CDCl_3$.

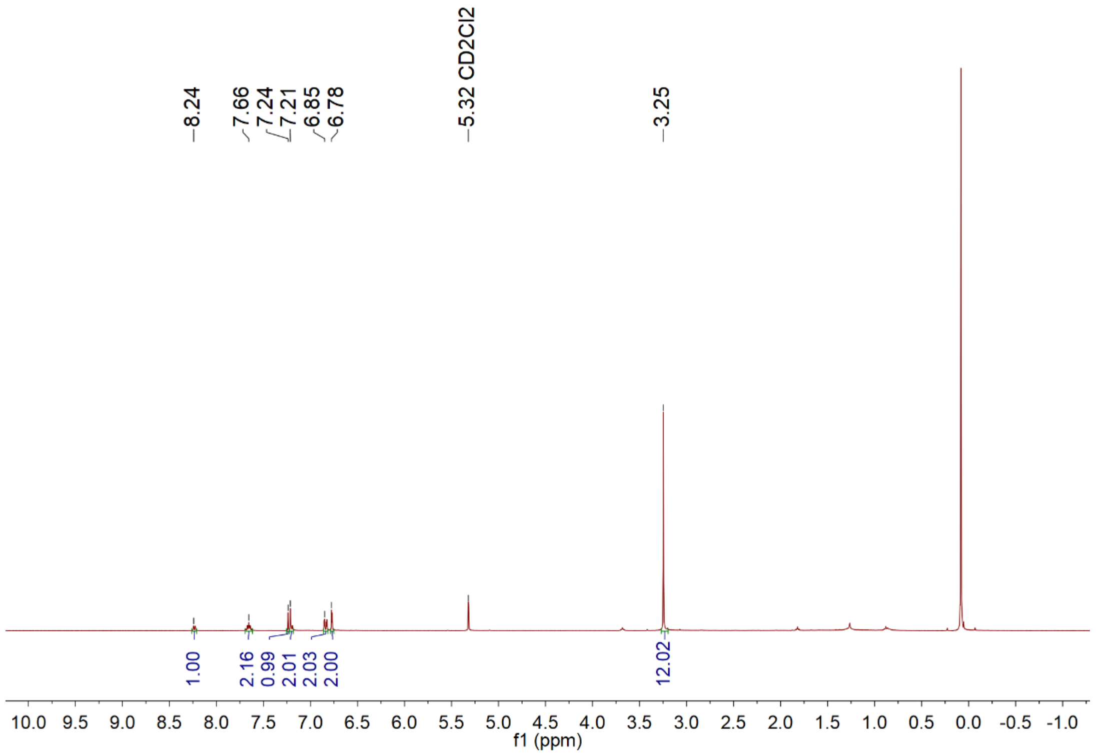

Figure S29. ^{1}H NMR spectrum of **3** in CD_2Cl_2.

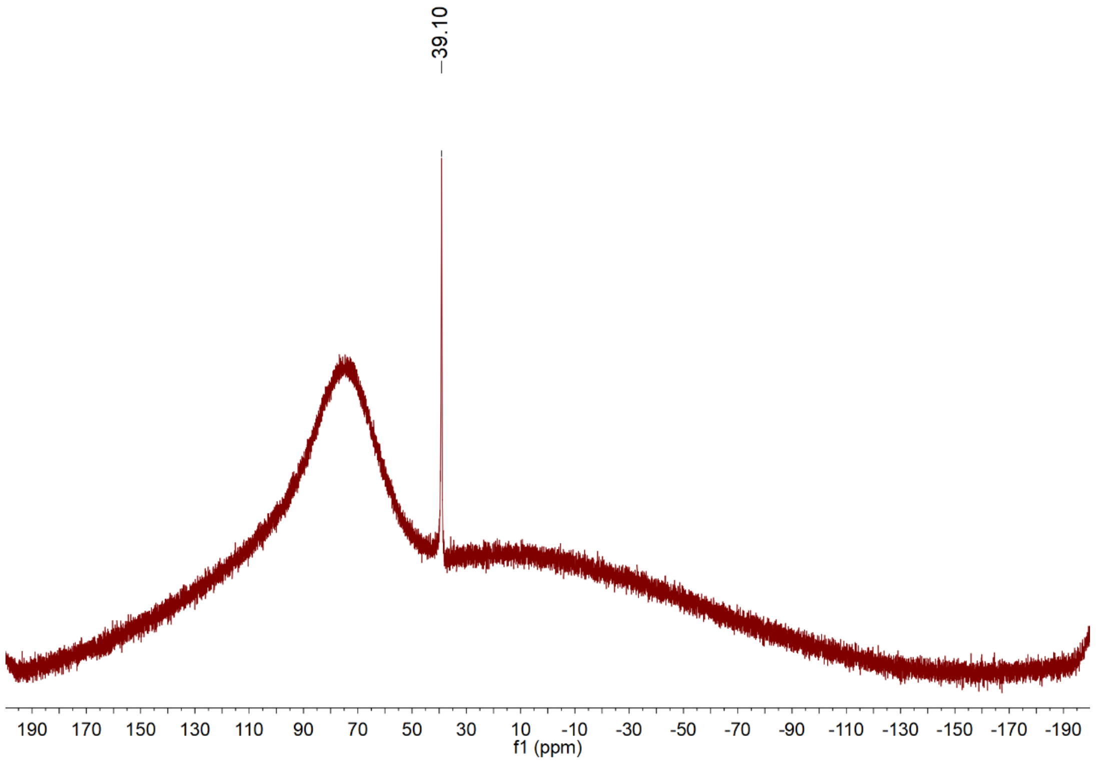

Figure S30. ^{27}Al NMR spectrum of **3** in CD_2Cl_2.

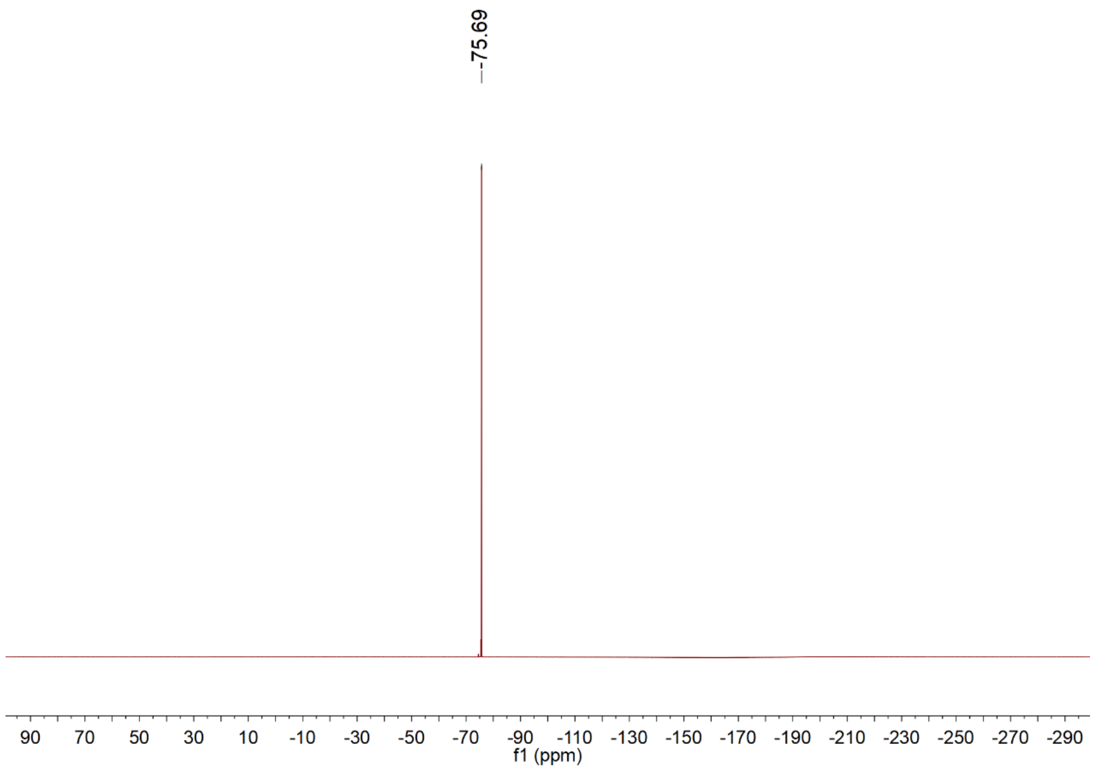

Figure S31. ^{19}F NMR spectrum of **3** in CD_2Cl_2.

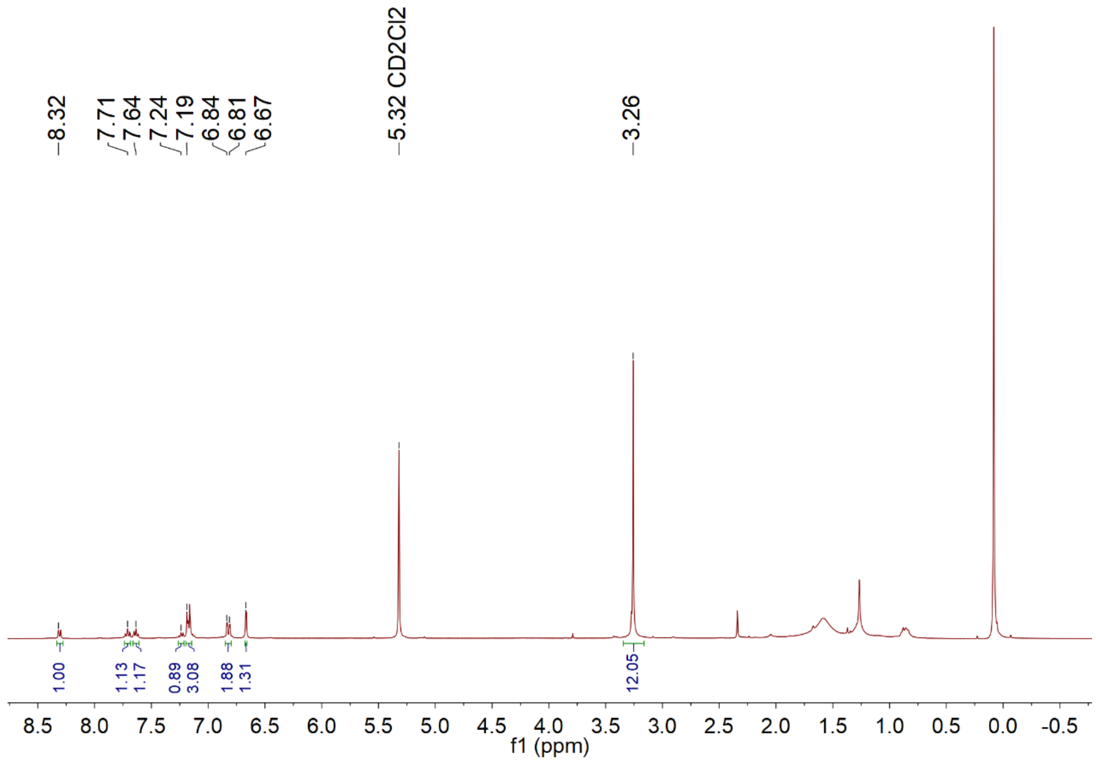

Figure S32. ^{1}H NMR spectrum of **4** in CD_2Cl_2.

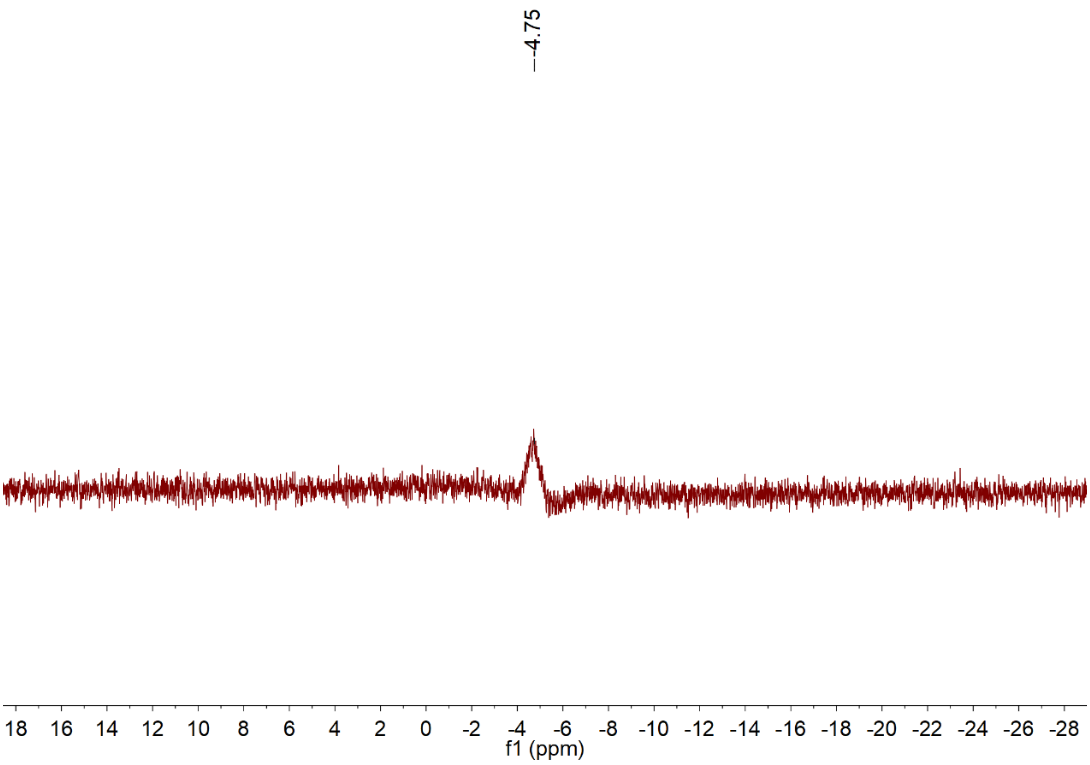

Figure S33. ^{11}B NMR spectrum of **4** in CD_2Cl_2.

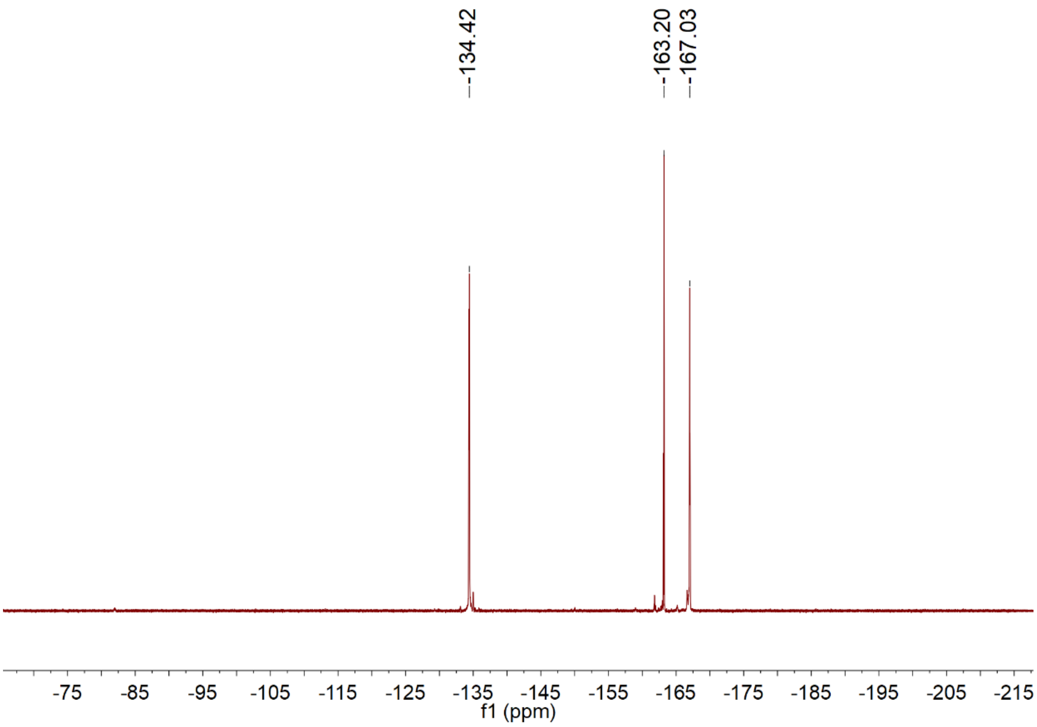

Figure S34. ^{19}F NMR spectrum of **4** in CD_2Cl_2.

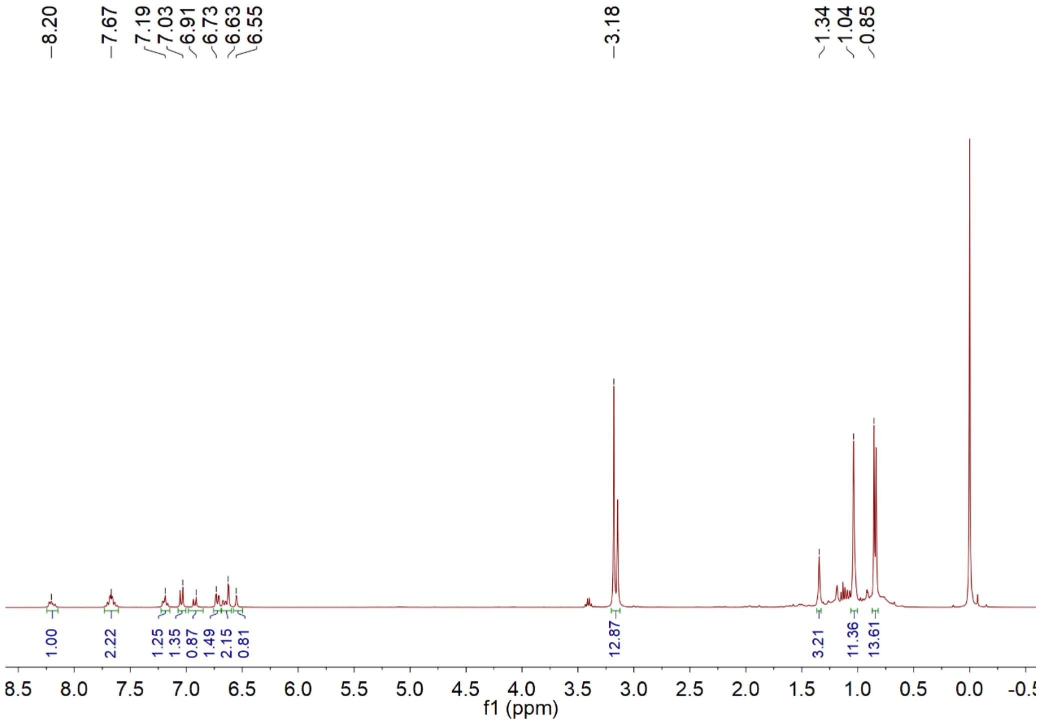

Figure S35. ^{1}H NMR spectrum of **5** in $CDCl_3$.

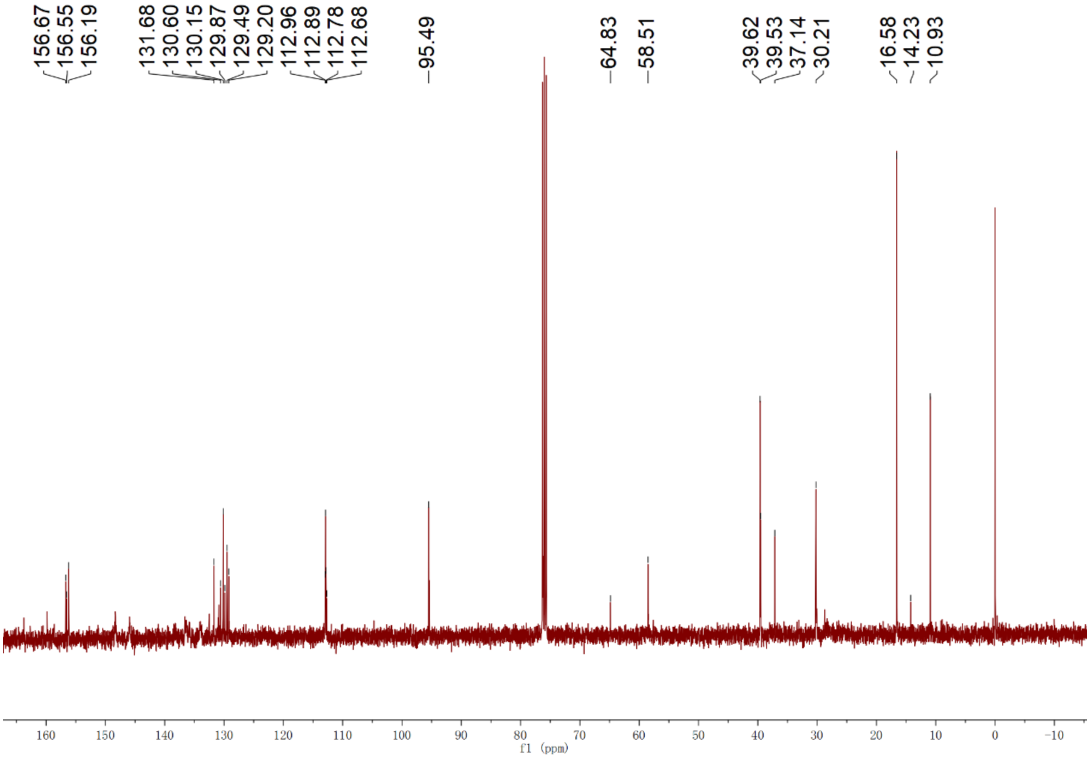

Figure S36. ^{13}C NMR spectrum of **5** in $CDCl_3$.

References

[49] Akita, S., Umezawa, N., Kato, N., and Higuchi, T. Array-based fluorescence assay for serine / threonine kinases using specific chemical reaction. *Bioorg. Med. Chem*. **2008**, *16*, 7788-7794.

[50] Huo, Y. Y., Ma, J. F., Han, L. J., Li, Y. P., Li, Z., Shi, Y. W., and Guo, W. Selective and sensitive visualization of endogenous nitric oxide in living cells and animals by a Si-rhodamine deoxylactam-based near-infrared fluorescent probe. *Chem. Sci.* **2017**, *8*, 6857-6864.

[64] Khalimon, A. Y., Piers, W. E., Blackwell, J. M., Michalak, D. J. and Parvez, M. A photo Lewis acid generator (PhLAG): controlled photorelease of $B(C_6F_5)_3$. *J. Am. Chem. Soc.* **2012**, *134*, 9601–9604.
